# Simultaneous enhancement of efficiency, stability and stretchability in binary polymer solar cells with a three-dimensional aromatic-core tethered tetrameric acceptor

**DOI:** 10.1093/nsr/nwaf019

**Published:** 2025-01-21

**Authors:** Yang Bai, Saimeng Li, Qingyuan Wang, Qi Chen, Ze Zhang, Shixin Meng, Yu Zang, Hongyuan Fu, Lingwei Xue, Long Ye, Zhi-Guo Zhang

**Affiliations:** State Key Laboratory of Chemical Resource Engineering, Beijing Advanced Innovation Center for Soft Matter Science and Engineering, Beijing University of Chemical Technology, Beijing 100029, China; College of Chemistry and Pharmaceutical Engineering, Huanghuai University, Zhumadian 463000, China; Tianjin Key Laboratory of Molecular Optoelectronic Sciences, Key Laboratory of Organic Integrated Circuits, Ministry of Education, Collaborative Innovation Center of Chemical Science and Engineering (Tianjin), Tianjin University, Tianjin 300350, China; State Key Laboratory of Chemical Resource Engineering, Beijing Advanced Innovation Center for Soft Matter Science and Engineering, Beijing University of Chemical Technology, Beijing 100029, China; State Key Laboratory of Chemical Resource Engineering, Beijing Advanced Innovation Center for Soft Matter Science and Engineering, Beijing University of Chemical Technology, Beijing 100029, China; State Key Laboratory of Chemical Resource Engineering, Beijing Advanced Innovation Center for Soft Matter Science and Engineering, Beijing University of Chemical Technology, Beijing 100029, China; State Key Laboratory of Chemical Resource Engineering, Beijing Advanced Innovation Center for Soft Matter Science and Engineering, Beijing University of Chemical Technology, Beijing 100029, China; State Key Laboratory of Chemical Resource Engineering, Beijing Advanced Innovation Center for Soft Matter Science and Engineering, Beijing University of Chemical Technology, Beijing 100029, China; State Key Laboratory of Chemical Resource Engineering, Beijing Advanced Innovation Center for Soft Matter Science and Engineering, Beijing University of Chemical Technology, Beijing 100029, China; Yaoshan Laboratory, Pingdingshan University, Pingdingshan 467000, China; Tianjin Key Laboratory of Molecular Optoelectronic Sciences, Key Laboratory of Organic Integrated Circuits, Ministry of Education, Collaborative Innovation Center of Chemical Science and Engineering (Tianjin), Tianjin University, Tianjin 300350, China; State Key Laboratory of Chemical Resource Engineering, Beijing Advanced Innovation Center for Soft Matter Science and Engineering, Beijing University of Chemical Technology, Beijing 100029, China

**Keywords:** tetrameric acceptor, efficiency, stability, stretchability, polymer solar cells

## Abstract

Polymer solar cells (PSCs) leverage blend films from polymer donors and small-molecule acceptors (SMAs), offering promising opportunities for flexible power sources. However, the inherent rigidity and crystalline nature of SMAs often embrittle the polymer donor films in the constructed bulk heterojunction structure. To address this challenge, we improved the stretchability of the blend films by designing and synthesizing a tethered giant tetrameric acceptor (GTA) with increased molecular weight that promotes entanglement of individual SMA units. The key to this design is using tetraphenylmethane as the linking core to create a three-dimensional and high C_2_ symmetry structure, which successfully regulates their aggregation and relaxation behavior. With GTA as the acceptor, its blend films with polymer donor PM6 exhibit significantly improved stretchability, with nearly a 150% increase in crack onset strain value compared to PM6:Y6. Moreover, the PSCs achieve an increased efficiency of up to 18.71% and demonstrate outstanding photostability, maintaining >90% of their initial power conversion efficiency after operating for over 1000 hours. Our findings demonstrate that by specifically designing three-dimensional tethered SMAs and aligning their molecular weights more closely with those of polymer counterparts, we can achieve enhanced stretchability without compromising morphological stability or device efficiency.

## Teaser

Film stretchability in polymer solar cells was enhanced by designing a tethered tetrameric acceptor with a 3D symmetric core, enabling effective control of aggregation and relaxation for better performance.

## INTRODUCTION

As a renewable energy technology, polymer solar cells (PSCs) have shown promising applications in wearable and portable electronic devices, as well as other fields, due to their light weight and flexible characteristics [1–3]. Recently, PSCs employing nonfullerene small-molecule acceptors (SMAs) [4,5] have achieved impressive power conversion efficiencies (PCEs) exceeding 19% [6–11]. Furthermore, ensuring robust device stability and durability is crucial for ensuring a prolonged operational lifetime and reliable performance under varying environmental circumstances [12].

As PSCs rely heavily on a bulk heterojunction (BHJ) structure to convert light into electricity, their performance in terms of mechanical and morphological stability, as well as device efficiency, originates from these BHJ structures. For the morphology of a BHJ structure in PSCs, it has been revealed that the diffusion behavior of SMAs follows Arrhenius behavior, and their diffusion coefficient decreases exponentially with an increase in the glass transition temperature (*T*_g_) [13,14]. Polymerizing or oligomerizing the SMAs has been proven to be a promising approach to increase the *T*_g_ of these materials [15–18]. This allows for kinetic control, inhibiting diffusion tendencies and retarding phase separation. As a result, the morphological stability of PSC devices is greatly improved [19–25].

As we know, the mechanical stability of PSCs is strongly determined by the intrinsic properties of the polymer donor, such as elasticity and viscosity. The viscoelastic nature of the polymer donor is primarily related to the relaxation properties of polymer chain motion. Under external stress, the mechanical response in the amorphous phase can be accommodated by the relative movement of adjacent segments in the polymer chain, or even the entire polymer chain, along the direction of the applied force [26,27]. This enables the polymer to maintain strength under stress [28–31]. Dynamic mechanical analysis (DMA) characterization reveals that polymer donors generally exhibit significant sub-*T*_g_ relaxation between −50°C and room temperature [32,33]. This indicates the potential for preparing stretchable PSC devices under ambient operating conditions. However, in such a BHJ structure, the high rigidity and crystalline nature of SMAs cannot be ignored, as they usually act as the breaking sites and become the bottleneck for stretchable applications. This embrittles the BHJ films, especially in highly conjugated SMA structures with high *T*_g_ [34]. Therefore, a significant challenge arises: how can we improve the mechanical stability and robustness of PSCs without compromising their morphological stability?

Fortunately, regulating the chemical structure and, consequently, the supramolecular interaction of the acceptor component offers a promising approach to achieving the desired robustness of blend films. This strategy fundamentally involves creating more free volume and strengthening the entanglement behaviors between polymer donor and SMA molecules for synergistic thermodynamic motion [35–37]. By optimizing the molecular organization of the acceptor component in the blend films, the sub-*T*_g_ relaxation of the polymer donor can be maintained, thereby preserving film robustness. Recently, this strategy has been demonstrated by incorporating oligomerized SMAs as a third component to cooperatively improve both morphological and mechanical stability [38–42]. Despite the significant potential of emerging oligomerization strategies, achieving a balance between efficiency, stability, and stretchability in PSCs remains a challenge, particularly in binary devices.

**Figure 1.**
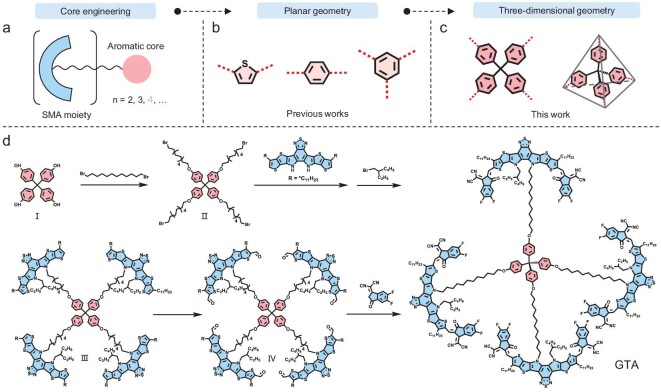
(a) General structure of tethered SMA molecules. The conceptual schemes of core engineering with (b) planar geometry and (c) three-dimensional geometry. (d) Synthetic route of GTA.

For the design of oligomers, differing from the classical strategy of connecting SMA units with rigid aromatic rings, we recently proposed an alternative strategy of tethering SMA units via flexible alkyl chains on an aromatic core (Fig. 1a). Initial studies suggest that this design can break the structural rigidity and stiffness of classical oligomeric acceptors, while maintaining higher *T*_g_ and crystallinity with a planar aromatic core. This achieves an efficiency >18.0% and retains 80% of initial efficiency over 1000 hours. Generally, increasing the number of repeating subunits in an oligomer, to achieve a molecular weight threshold resembling its polymer counterpart, can enhance physical properties by promoting greater intermolecular entanglement and interactions, thereby improving mechanical properties [43,44]. This would promote greater intermolecular entanglement and interaction for enhancing those properties. In this context, it becomes intriguing to further increase the SMA-subunits and create more free volume inside the acceptor materials. However, this approach poses challenges by introducing complexity and loosening molecular packing, which affects the electronic state coupling of the SMA subunits and, consequently, reduces device efficiency.

In this regard, we designed a tethered tetrameric SMA using tetraphenylmethane (TPM) as the linking core. In contrast to our previous designs featuring a planar aromatic core (Fig. 1b) [45,46], the cross-junction structure of the TPM core introduces a three-dimensional geometry (Fig. 1c), which is expected to increase free volume in the aggregated state within films. Moreover, the highly symmetrical structure of the TPM core effectively governs the molecular packing of SMA subunits and ensures the appropriate crystallinity of the acceptors. Significantly, its blend films with a benchmark polymer donor PM6 demonstrate favorable relaxation properties, showing nearly a 150% increase in crack onset strain value compared to benchmark PM6:Y6 counterparts. Also, this device achieves a remarkable photostability, maintaining >90% from the initial power conversion efficiency (PCE) of 18.71% after continuous operation for more than 1000 hours. Notably, this is the first case of a nonfullerene acceptor enabling improved efficiency, stability and stretchability in binary polymer solar cells.

## RESULTS AND DISCUSSION

### Materials synthesis and characterization

**Figure 2.**
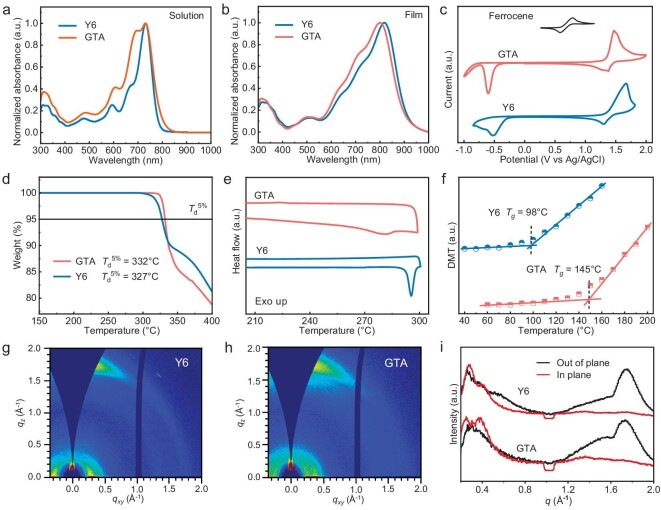
Normalized UV-vis absorption spectra of the GTA and Y6 (a) in chloroform and (b) as thin film. (c) Cyclic voltammogram of the GTA and Y6 films. (d) TGA curve of GTA with a heating rate of 20 K min^−1^. (e) DSC thermograms of the GTA and Y6 with a heating rate of 10°C min^−1^ under nitrogen atmosphere. (f) The deviation metric obtained from the normalized absorption spectra of thin films at varied temperatures, with the corresponding *T*_g_ values inserted. The 2D GIWAXS diffraction patterns of (g) Y6 films and (h) GTA films, and (i) the line cuts curves.

The synthetic routes of the tetrameric acceptor GTA are illustrated in Fig. 1d, following a procedure outlined in our recent publication [45], with the total yield of ∼54%. Notably, GTA molecules were quantitatively obtained via a BF_3_·OEt_2_-catalyzed Knoevenagel condensation method developed by our group [47]. UV-vis absorption spectra of the studied materials in chloroform and as thin films are presented in Fig. 2a and b. In dilute solution, GTA and Y6 exhibit similar main absorption peaks centered around 732 nm. Additionally, similar to the dimers described in our previous work [46], GTA shows a much stronger shoulder absorption peak around 694 nm, originating from Davydov splitting induced by its intrinsic folded geometry in dilute solution. In solid films, with a flexible chain regulating the packing of SMA units, the main absorption peak of GTA displays a noticeable hypsochromic shift of ∼18 nm compared to that of Y6. The relatively enhanced intensity of the shoulders of the Davydov splitting peaks (around 720 nm) and broader absorption spectra also indicate additional aggregation in GTA films bearing a closer distance of the tethered molecules.

The frontier orbital energy levels of GTA and Y6 were estimated through electrochemical cyclic voltammetry (CV) measurements, as depicted in Fig. 2c and summarized in Table 1. GTA exhibits a larger band-gap and a significantly higher lowest unoccupied molecular orbital energy level (*E*_LUMO_) value (−3.87 eV versus −4.09 eV for Y6), which favors a higher open-circuit voltage (*V*_oc_) in devices. According to Fig. 2d, GTA exhibits slightly better thermal stability than Y6, with ∼5°C enhancement in thermal decomposition temperature (*T*_d_) (5% weight loss). From differential scanning calorimetry (DSC) measurements (Fig. 2e), GTA exhibits a significantly decreased lower melting enthalpy (Δ*H*_m_) of 9.41 *J* g^–1^, compared with Y6. Additionally, the *T*_g_ of GTA was estimated to be 145°C (Fig. 2f, Fig. S1), markedly higher than the monomeric Y6 at ∼98°C. This demonstrates that the larger molecular weight of the GTA molecule does indeed contribute significantly to the increase in *T*_g_. The solubility of the Y6 and GTA in chloroform were measured based on the Beer–Lambert law [48], to be 37.9 mg mL^−1^ and 28.4 mg mL^−1^, respectively, as shown in Fig. S2.

**Table 1.** The physicochemical and aggregated properties of GTA and Y6.

						CL	CL						
	*λ*_max_	*λ*_max_	*E*_g_^opt^	*E*_LUMO_	*E*_HOMO_	[100]	[010]		*T_m_*	Δ*H*_m_		χ	*μ_e_*
Acceptors	(nm)^a^	(nm)^b^	(eV)^c^	(eV)^d^	(eV)^d^	(Å)^e^	(Å)^e^	*r*DoC ^f^	(°C)	(*J g*^−1^)	*T*_g_ (°C)^g^	(PM6)	(10^–4^ cm^2^ v^−1^s^−1^)
Y6	732	819	1.348	−4.09	−5.70	43.84	30.73	0.94	295.5	28.15	99	0.89	1.46
GTA	731	801	1.362	−3.87	−5.73	64.26	35.57	1.00	282.6	9.41	145	0.99	8.02

a Solution

b Film

c Calculated from the absorption edge of the films: *E*_g_^opt.^ = 1240/*λ*_edge_

d Calculated according to the equation: *E*_LUMO/HOMO_ = −e (*φ*_red/ox_ +4.366) (eV)

e Calculated from the Scherer equation: CL = 2π*K*/Δ*q*, where Δ*q* is the full-width at half-maximum of the peak and *K* is a shape factor (0.9 was used here)

f Calculated the relative degree of crystallinity (*r*DoC) by integrating the scattering intensity of the lamellar diffraction peaks

g Estimated with absorption spectroscopy

The crystallization behavior of GTA molecules was investigated using grazing-incidence wide-angle X-ray scattering (GIWAXS), as depicted in Fig. 2g–i and Fig. S3. It can be seen that both the Y6 and tetramer in neat films favor a face-on orientation relative to the substrate, as revealed by the lamellar stacking peaks (100) in the in-plane (IP) direction and the strong π-π stacking peaks (010) in the out-of-plane (OOP) direction. For the coherence length (CL) of the (100) peaks and (010) peaks, GTA shows simultaneous increased values, with respective values of 64.3 Å and 35.6 Å, relative to those of Y6 (43.8 Å and 30.7 Å). Furthermore, by analyzing the azimuthal distributions of (010) diffraction peaks, pole figures were obtained for quantitative comparison. The relative degrees of crystallinity (*r*DoC) of Y6 and GTA films were calculated to be 0.94 and 1.00, respectively. This indicates a relatively higher degree of crystallinity for GTA with a high C_2_ symmetry of the aromatic linker, which may partially contribute to the significantly higher electron mobility (*μ*_e_) of GTA (8.02 × 10^–4^ cm^2^ V^−1^ s^−1^) compared to Y6 (1.46 × 10^–4^ cm^2^ V^−1^ s^−1^), as shown in Fig. S4.

### Photovoltaic performance

**Figure 3.**
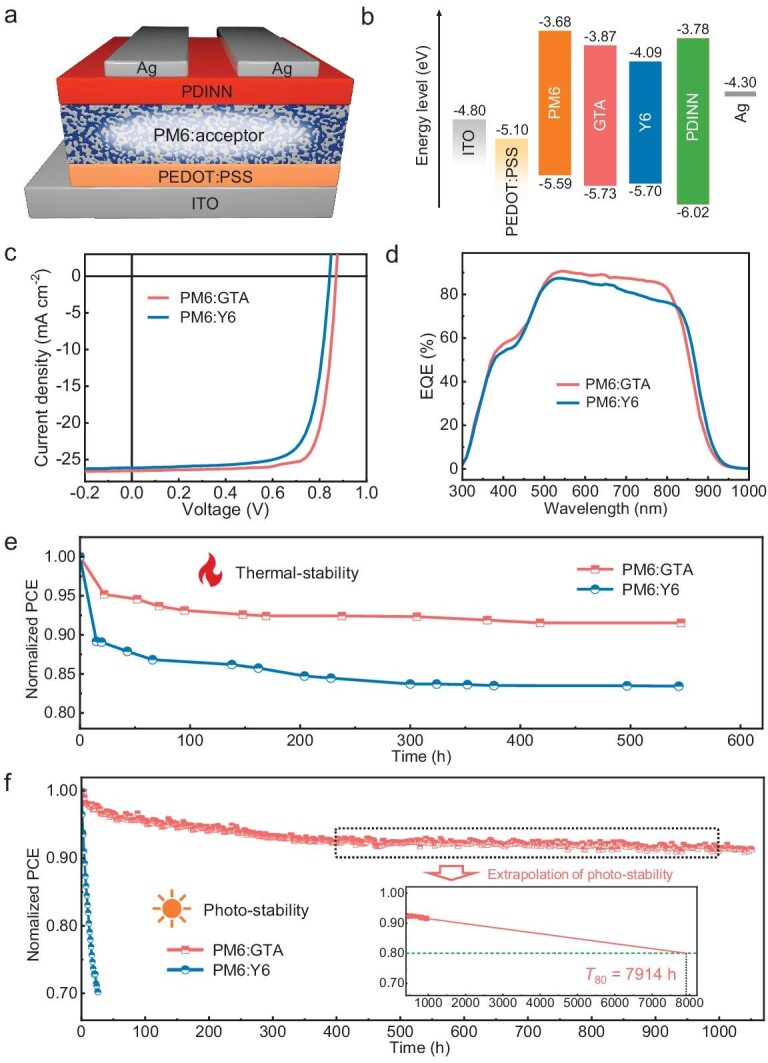
(a) Device structure of the PSCs. (b) Energy level diagram of the device. (c) *J-V* curves of the best PSCs with D:A weight ratio of 1:1.2 under the illumination of AM 1.5 G, 100 mW cm^−2^ and (d) EQE spectra of corresponding PSCs. (e) Normalized PCEs of the PM6:Y6- and PM6:GTA-based devices under long-term annealing at 100°C in a nitrogen-filled glovebox. (f) MPP stability test of the PM6:Y6- and PM6:GTA-based devices under 1-Sun equivalent illumination from white LEDs at MPP conditions in open-air.

PSC devices are fabricated with a conventional architecture (Fig. 3a and b), and conjugated polymer PM6 is chosen as the benchmark donor, and PDINN (aliphatic amine-functionalized perylene-diimide) is used as the cathode interlayer facilitating carrier collection. The current-density-voltage (*J-V*) curves are shown in Fig. 3c, and photovoltaic parameters of the devices are collated in Table 2. The control devices based on monomeric Y6 possess a typical PCE of 16.31% with a *V*_oc_ of 0.841 V, a *J*_sc_ of 26.13 mA cm^−2^, and an FF of 74.21%. In contrast, the PM6:GTA–based devices show improved photovoltaic performance, with a *V*_oc_ of 0.882 V, a *J*_sc_ of 26.64 mA cm^−2^, an FF of 79.64%, and finally achieve a much higher PCE of 18.71%. Moreover, the efficiency is the highest reported for those oligomeric accepters with flexible chains (Fig. S5 and Table S1). To confirm the *J*_sc_ of the devices, external quantum efficiencies (EQEs) were conducted using the AM 1.5 G solar spectrum (Fig. 3d), showing a discrepancy of <5%.

**Table 2.** Photovoltaic parameters of the PSCs based on PM6:acceptor with D:A weight ratio of 1:1.2 and thermal annealing at 100°C for 5 min. under the illumination of AM1.5 G, 100 mW cm^−2^.^a^

					*μ_h_*	*μ_e_*	
Active layer	*V*_oc_ (V)	*J*_sc_ (mA cm^−2^)	FF (%)	PCE (%)	(10^–4^ cm^2^v^−1^s^−1^)	(10^–4^ cm^2^v^−1^s^−1^)	*μ_h/_μ_e_*
PM6:Y6	0.841(0.839 ± 0.003)	26.13(26.50 ± 0.60)	74.21(73.60 ± 0.70)	16.31(15.80 ± 0.40)	3.19	4.70	1.47
PM6:GTA	0.882(0.876 ± 0.005)	26.64(26.41 ± 0.13)	79.64(79.32 ± 0.31)	18.71(18.64 ± 0.06)	4.92	5.13	1.04

a Average values based on ten devices.

To quantitatively investigate the charge transport properties of the GTA-based devices, carrier mobilities are calculated by measuring the single-carrier devices using the space charge limited current (SCLC) method. As presented in Fig. S6 and Table 2, the hole/electron mobilities (*μ*_h_/*μ*_e_) of the GTA-based blend film were 4.92 × 10^–4^ cm^2^ V^−1^ s^−1^/5.13 × 10–4 cm^2^ V^−1^ s^−1^ with a balanced ratio of 1.04. This represents a significant improvement compared to Y6-based blend film (3.19 × 10^–4^ cm^2^ V^−1^ s^−1^/4.70 × 10–4 cm^2^ V^−1^ s^−1^, ratio of 1.47) and the planar TDY-α–based blend film [46] (5.88 × 10^–4^ cm^2^ V^−1^ s^−1^/4.92 × 10^–4^ cm^2^ V^−1^ s^−1^, ratio of 1.19, Fig. S7). The higher and more balanced charge transport behaviors indicated that the cross-junction structure of GTA facilitates entanglement within SMA subunits and between GTA and PM6 molecules. This enhanced structural interaction potentially creates additional charge carrier channels, reducing charge accumulation and recombination, ultimately leading to a much higher FF of the devices.

To gain insights on exciton dissociation and charge collection behavior of the PSCs, the charge dissociation probability (P(E, T)) is estimated by the relationship between photo current density (*J*_ph_) and the effective voltage (*V*_eff_) of the devices (Fig. S8). Under short-circuit conditions, the GTA-based devices have higher P(E, T) values >99.1%, which is higher than that of the Y6-based device (95.2%). The result demonstrates an improvement of exciton dissociation and charge collection process for the GTA. Furthermore, the charge recombination behaviors are investigated by the dependence of *V*_oc_ and *J*_sc_ on light intensity (*P*_light_). As shown in Fig. S9, for the relationship between *V*_oc_ and *P*_light_, the slopes of the Y6- and GTA-based devices are 1.17 *k*T/*q* and 1.02 *k*T/*q*, respectively. The smaller slope indicates that the GTA-based devices possess less trap-assisted recombination. On the other hand, the relationship between *J*_sc_ and *P*_light_ can be expressed as *J*_sc_ ∝ (*P*_light_)^α^, which reveals charge bimolecular recombination in devices. The fitting α values of the Y6- and GTA-based devices are 0.97 and 0.99, respectively. The highest α value (closer to 1) demonstrates that there is effective carrier collection and negligible bimolecular recombination in the GTA-based devices under short-circuit conditions. The above results are in line with a higher *J*_sc_ and FF value of the GTA-based device.

### Optimized device stability

We initially investigated device stabilities under dark conditions in a nitrogen-filled glovebox with thermal stress (Fig. 3e). As anticipated, the two devices exhibited dramatically different efficiency losses over time, with the GTA-based device showing a PCE decrease of only 5% compared to a 15% loss for the Y6-based device after 500 hours. Subsequently, we subjected encapsulated devices to air exposure under 1-Sun equivalent illumination from white LEDs at maximum power point (MPP) conditions (Fig. 3f). The GTA-based devices demonstrated outstanding photostability, maintaining >90% of their initial PCE after 500 hours of operation, whereas the PCE of the Y6-based device dropped by 60% within 20 hours. Ultimately, the photostability of GTA-based devices was tested over 1000 hours, and the corresponding linear extrapolation of the original data between 400 and ∼1000 hours suggested an average T_80_ lifetime of ∼8000 hours, as shown in Fig. S10.

The morphological stability under light or heat is kinetically controlled when using acceptors with high *T*_g_, where their diffusion into the donor polymer follows an Arrhenius behavior [14,49]. Due to GTA's significantly higher *T*_g_ (145°C) compared to Y6 (98°C), the diffusion coefficients (*D*_85_) for the respective blend films were calculated as 6.90 × 10^–21^ and 6.85 × 10^–18^ cm^2^ s^−1^, respectively. The *D*_85_ value of the GTA-based blend film is three orders of magnitude lower than that of Y6, effectively delaying acceptor diffusion into the polymer domain [14].

The higher stability of the GTA-based device can be understood in terms of thermodynamics, specifically through the Flory–Huggins interaction parameter (χ) of blend films. The χ value was evaluated via the *T*_m_ depression method for acceptors in homogeneous PM6 mixtures with various weight ratios (Fig. S11). According to the Ade-O'Connor-Ghasemi framework [14], the PM6:Y6 blend morphology is neither thermodynamically nor kinetically stabilized, exhibiting a hypo-miscible status. An appropriate increase in χ can enhance morphological stability. Proper donor-acceptor miscibility is necessary because excessively low miscibility (higher χ) over-purifies mixed domains, while excessively high miscibility (lower χ) results in insufficient phase separation, both leading to performance deterioration. Our calculations show χ values of 0.89 for PM6:Y6 and 0.99 for PM6:GTA blends. The higher χ value for the GTA-based blends indicates a more hypo-miscible system, resulting in suppressed diffusion-enabled demixing of the morphology.

### Aggregation behavior and morphology

**Figure 4.**
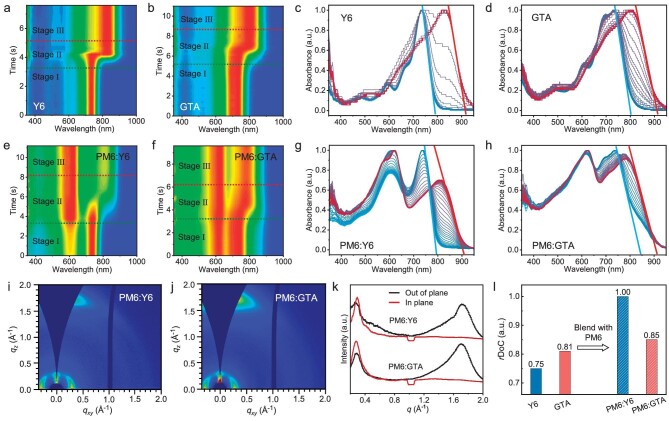
Analysis of *in-situ* UV-vis spectrum of spin-coated films of Y6, GTA and the corresponding blends with PM6. (a, b, e, f) The color mapping of absorption spectra as a function of spin-coating time, and (c, d, g, h) the corresponding stacking plots. (i, j) The 2D GIWAXS diffraction patterns of the blend films, and (k) the line cuts curves. (i) The corresponding *r*DoC values calculated via the azimuthal distributions of (010) diffraction peaks of both the pristine acceptor films and blend films.

With a tethered structure, we were interested in their aggregation behavior and their influence on morphology. Therefore, we monitored the spectral evolution during the film-forming process using *in-situ* UV-vis spectroscopy, as depicted in Fig. 4a–h. The two dashed lines in the color mapping of absorption spectra divide the spectral evolution into three stages: the solution stage (I), phase transition stage (II) and film stage (III) [50–52]. For stage II, the main peaks as a function of film-forming time are depicted in Fig. S12, allowing for a detailed comparison of their phase transition behaviors. The stacking plots of absorbance, provided in Fig. 4c–d, and g–h, clearly show the progressive evolution of the absorption edge from the initial solution state (marked in blue) to the final film state (marked in red).

For the neat films of acceptors, Fig. 4a and b show the evolution of the absorption profile of the pristine acceptors over time. Compared to Y6, the tetrameric GTA demonstrates a slower and more delayed liquid-solid transition process. It exhibits a phase transition time approximately twice as long as that of Y6 films (3.8 seconds to 2.0 seconds, Fig. S12a, b). The prolonged phase transition can be primarily attributed to the entanglement of individual SMAs in the tetramer, which impedes their packing and requires more time to organize into a solid film.

For the blend films we focus on the acceptor component, thus we tracked the evolution of the major absorption peak around 750–800 nm. As the solvent evaporates, the Y6 blend film exhibits a noticeable red shift in the absorption edge along with a sharp decrease in peak intensity (Fig. 4g). The transition edge occurs around 5 seconds, with a corresponding phase transition time of 5.2 seconds in stage II. In contrast, the absorption edge of the tetramer blend film progressively red-shifts, with the edge line remaining almost parallel throughout the process and without significant variation in peak intensity (Fig. 4h). The corresponding phase transition time is notably reduced to just 3.6 seconds.

For the PM6:GTA–solution, in addition to the potential entanglement of individual SMAs within the tetramer, there is also the possibility of entanglement between individual SMAs and the polymer donor chains. Due to the rod-like structure of the polymer chains, in solution, chain entanglement between the polymer donor and the tethered acceptor can form a network with high molecular weight, decreasing solubility in the solvent. As a result, these entangled molecules tend to precipitate out of the solution more readily. This accelerates the nucleation and growth of solid domains as the solvent evaporates, leading to a faster transition from liquid to solid. This reduces the distance and time required for the molecules to diffuse and organize into a solid film. Consequently, this enhanced interaction and organization can lead to a quicker phase transition from liquid to solid.

The influence of three-dimensional tethered tetrameric structure on molecular packing and crystal texture in the blend films was investigated by GIWAXS measurement, as shown in Fig. 4i–k and Fig. S14. The PM6:GTA–blend films exhibit decreased CL values of lamellar stacking (54.90 Å) and π-π stacking (30.1 Å) than those of PM6:Y6–blend films (65.8 Å, 36.0 Å), and the *r*DoC values of PM6:Y6 and PM6:GTA–blend films were found to be 1.00 and 0.85, respectively (Fig. 4l, Fig. S15). Interestingly, the reverse tendency of CL and *r*DoC values of the blend films relative to that of pristine acceptor films suggests that blending GTA molecules affects the overall aggregation behavior, likely due to specific intermolecular entanglements with polymer donors.

Meanwhile, AFM and TEM were carried out to elucidate the morphological characteristics for a deeper understanding between aggregated behaviors and phase separation features. As shown in Fig. S16, the root-mean-square roughness (*R*_q_) of PM6:GTA films (1.23 nm) is relatively larger than that of PM6:Y6 films (1.10 nm). More importantly, the AFM phase images and TEM images of PM6:GTA films show the more desired fibrous morphology. The fundamental and phenomenological results above synchronously indicate the unique aggregation behaviors of molecules in PM6:GTA films, which may have potential influences on their mechanical properties.

### Improved mechanical robustness

**Figure 5.**
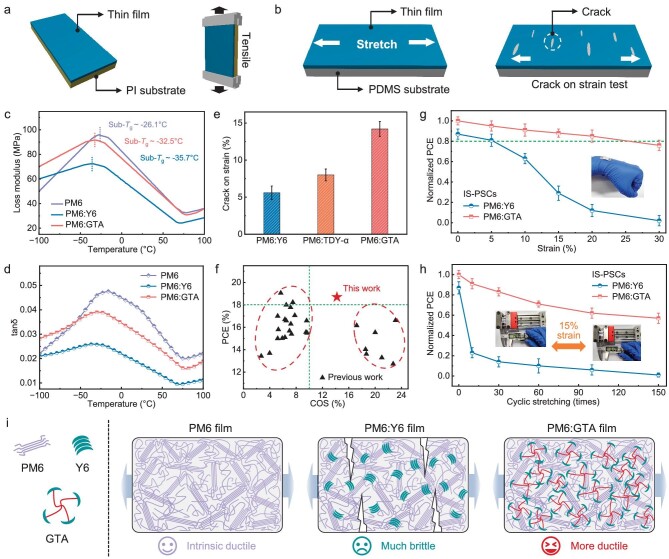
The illustration of (a) DMA and (b) FOE method for characterizing the tensile properties of thin films. The comparison of curves of (c) loss modulus and (d) tanδ versus temperature of PM6, PM6:Y6 and PM6:GTA. (e) The crack onset strain (COS) of the blend films. (f) The statistical PCE and COS values of binary PSCs in our work and those reported. (g) Plots of normalized PCE values of intrinsically stretchable PSCs under various strains. (h) Plots of normalized PCE values of intrinsically stretchable PSCs with stretching cycles under 15% strain. (i) Schematic illustration of the morphological features and crack states of PM6, PM6:Y6 and PM6:GTA films under external stress.

To investigate whether the specific aggregation behaviors of GTA-based blend films influence their thermomechanical properties, we conducted dynamic mechanical analysis (DMA) on pristine PM6, as well as the corresponding Y6- and GTA-based blend films, as illustrated in Fig. 5a. The loss modulus and storage modulus curves are shown in Fig. S17. As is well known, the storage modulus indicates the material's ability to store energy under stress, reflecting its rigidity. In contrast, the loss modulus represents the energy dissipation capability of the material. The sub-*T*_g_ loss tangent (tanδ) was calculated as the ratio of the loss modulus to the storage modulus [32,33]. Notably, at temperatures below the *T*_g_, most polymer donors exhibit significant thermomechanical relaxation processes, referred to as secondary thermal relaxations or sub-*T*_g_ relaxations [53]. These sub-*T*_g_ relaxations occur in the amorphous phase and are associated with the motions of minor molecular moieties, which correspond to lower activation energies compared to the primary relaxation processes occurring above *T*_g_.

Generally, the *T*_g_ can be obtained conventionally as either the peak temperature of tanδ or the peak temperature of the loss modulus, and so do the sub-*T*_g_ values. As shown in Fig. 5c and Fig. S18, the PM6 neat films show a sub-*T*_g_ peak ∼−26.1°C. While with the blend with Y6, the sub-*T*_g_ peak significantly reduces to ∼−35.7°C, validating that the high rigidity and crystalline nature of SMAs will distinctly suppress the sub-*T*_g_ relaxation of PM6 [54–57]. Notably, the GTA-based blend exhibits a relatively higher sub-*T*_g_ peak at −32.5°C than that of the Y6-based blend, indicating that the unique structure of GTA may increase the entanglement of the blend materials and the critical activation volume for molecular motions, allowing for energy dissipation through molecular rearrangement or inter/intramolecular slip and friction.

Moreover, there are drops in the sub-*T*_g_ tanδ intensity in both blend films compared with PM6 films, consistent with other research findings [33,58]. The strength of the sub-*T*_g_ relaxation was quantified by integrating sub-*T*_g_ tanδ over the temperature range, resulting in the sub-*T*_g_ relaxation strength (*I*_tanδ_), as depicted in Fig. 5d and Fig. S19. The *I*_tanδ_ values for PM6, PM6:Y6 and PM6:GTA films were calculated to be 6.74, 3.78 and 5.83, respectively. Compared to the PM6:Y6–blend film, the significantly higher *I*_tanδ_ value of the PM6:GTA film indicates a relatively smaller suppression of sub-*T*_g_ relaxation, and weaker inhibition of the free volume and energy barrier for polymer chain movement. According to Eyring theory, this would have a positive effect on decreasing the film's yield stress and potentially increasing its elongation at breaking point. In conclusion, the blending of GTA molecules could theoretically retain the mechanical properties of the PM6 film to a greater extent.

Thus, the mechanical properties of the blend films were evaluated using the ‘film-on-elastomer’ (FOE) tensile test method (Fig. 5b). As depicted in Fig. 5e, the neat PM6:Y6 film possesses the COS value of 5.6 ± 0.9%. While the blend of GTA results in a softened film, with a significant increased COS value of 14.2 ± 1.0%, which is also higher than that of planar TDY-α–based blend films (8.0 ± 0.8%). Meanwhile, the elastic moduli were also analyzed according to a buckling-based method (Fig. S20), revealing an elastic modulus of 1.54 ± 0.06 GPa for PM6:Y6 film and a decreased elastic modulus of 1.42 ± 0.12 GPa for PM6:GTA film. These values align with the paracrystalline disorder factor (*g*-factor) of the (010) peaks of PM6:Y6 films (12.7%) and PM6:GTA films (14.0%). The nearly 150% improvement of COS value and smaller elastic modulus confirm that the stretchability of the GTA-based blend film is indeed improved, thanks to the design of three-dimensional tethered acceptors. We have summarized the reported PCE versus COS values for binary PSCs in Fig. 5f and Table S2. It can be seen that our device, utilizing a tetrameric acceptor, well balances efficiency and mechanical properties.

Flexible devices with high mechanical ductility have attracted considerable attention, thus, we fabricated flexible PSC (F-PSC) devices with the same device architecture as the rigid devices, replacing the ITO glass electrode with the PET. Remarkably, the GTA-based F-PSC devices achieved an enhanced PCE of 16.43% relative to that (14.45%) of a Y6-based control device (Fig. S21 and Table S3). Furthermore, intrinsically stretchable PSC (IS-PSC) devices were also fabricated to investigate mechanical robustness. Stretch-release tests of the IS-PSCs under various strains revealed that under 15% stretch, GTA-based devices retained 88% of the initial value, nearly 3 times that of the Y6-based device (29%), indicating improved stretching tolerance (Fig. 5g). Furthermore, cyclic stretch-release tests for IS-PSC devices at 15% strain showed that, GTA-based devices retained ∼76% of its initial PCE value after 150 cycles, while the Y6-based device was almost damaged (Fig. 5h).

To better understand mechanical robustness, we propose a conceptual illustration in which the molecular chains of the polymer donor resemble a bundle of stretchable ropes under external stress (Fig. 5i). The presence of the tetrameric acceptor GTA featuring flexible alkyl chains and three-dimensional geometry allows it to act as a multi-branched structure that binds several PM6 chains together, thereby enhancing the intermolecular entanglement density. This network of entangled polymer chains effectively suppresses the sub-*T*_g_ relaxation. Consequently, the mechanical properties of PM6 blend films are significantly enhanced, maintaining their ductility. In contrast, the addition of stiff Y6 behaves like inserting rigid bricks, which restricts the stretching of the ‘polymer springs’ and impedes molecular motion. This structural rigidity limits the formation of a robust network of intermolecular entanglements, thereby reducing the material's flexibility and ductility. The three-dimensional geometry of GTA plays a crucial role in enhancing the network's connectivity and stability.

## CONCLUSION

In summary, we attempt to resolve the contradiction of the morphological stability and mechanical robustness of polymer solar cells, with a novel design of tetrameric acceptor GTA based on a tethered strategy. The three-dimensional geometry of the tetraphenylmethane core produced tetrameric acceptor GTAs which can not only effectively realize the higher *T*_g_ (145°C) for long-lived operation stability, but also collaboratively regulate their ordered aggregation, and the entanglements with polymer donors, which improve the mechanical robustness of binary blend films. Generally, employing high *T*_g_ acceptors typically results in improved morphological stability at the expense of mechanical stability. While for our case, the much higher *I*_tanδ_ value of PM6:GTA–film indicates the relatively smaller sub-*T*_g_ suppression strength and more desired relaxation properties, which shows potential benefit for decreasing the film's yield stress and increasing the film's elongation at breaking point. Thus, the PM6:GTA–based blend films exhibit a nearly 150% improvement in the COS value compared to the PM6:Y6 counterpart. As a consequence, the GTA-based IS-PSCs could retain 88% of the initial PCE value under 15% stretch and ∼76% after 150 stretch-release cycles, whereas the Y6-based devices failed. Moreover, the PM6:GTA–based devices gain a much higher PCE of 18.71%, and perform outstanding photo-stability that maintain >90% of initial PCE after operating for over 1000 hours. Notably, this is to date the only molecular design approach to simultaneously improve the efficiency, stability and stretchability of binary polymer solar cells. Our work highlights the potential advantages of our unique tethered approach, especially with the design of a three-dimensional linking core that increases free volume and entanglement with the polymer donor. Through this unique design, we carefully regulated the aggregation and relaxation properties, thereby simultaneously enhancing the efficiency, morphological stability and mechanical properties of PSCs. Further exploration of this molecular architecture can continually advance the practical applications of flexible PSCs.

## Supplementary Material

nwaf019_Supplemental_File
