# Supplementary material for: Simultaneous enhancement of efficiency, stability and stretchability in binary polymer solar cells with a three-dimensional aromatic-core tethered tetrameric acceptor
Source: Natl Sci Rev. 2025 Jan 21;12(3):nwaf019. doi: 10.1093/nsr/nwaf019 (PMC11841366; doi:10.1093/nsr/nwaf019)
Supplement: nwaf019_Supplemental_File [file nwaf019_supplemental_file.pdf]

Supplementary information

Materials Synthesis

Reagents and materials: Polymer donor PM6 was purchased from Solarmer Materials. Y6 and 2-(5,6-difluoro-3-oxo-2,3-dihydro-1H-inden-1-ylidene)malononitrile (2F-IC) were purchased from eFlexPV. C_{11}TT(N-H)BT (Y6-core) was purchased from Hyper, Inc. The other reagents and chemicals were purchased from Bidepharm Co. Ltd (Shanghai, China) and J&K Scientific Co. Ltd (Beijing, China) and were of analytical grade and used without further purification. The GTA molecule was synthesized according to the detailed routes in **Scheme S1**.

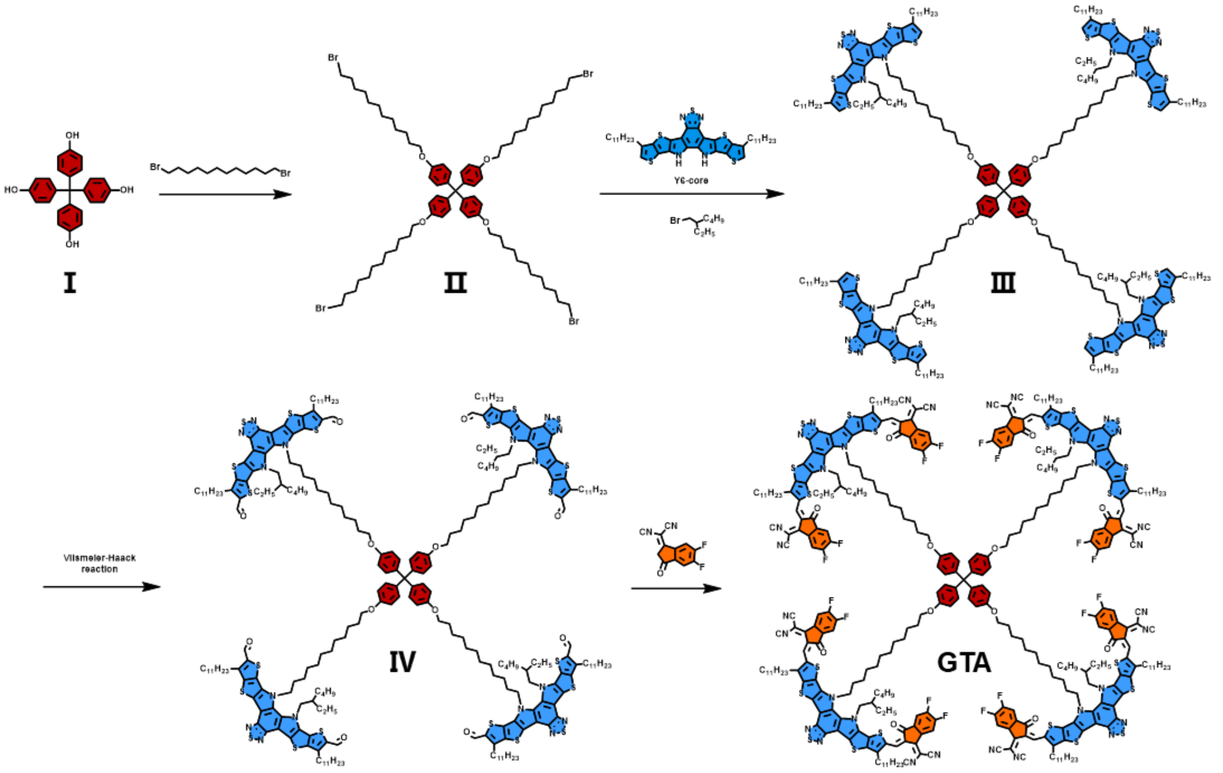

Scheme S1 Synthetic routes of GTA

Synthesis of compound Ⅱ: Compound Ⅰ (tetrakis(4-hydroxyphenyl)methane, 1.0 mmol), 1,12-Dibromododecane (10.0 mmol) and K_2CO_3 (10.0 mmol) were mixed in acetonitrile (20.0 ml) to stir at 60℃ for 24 h under argon atmosphere. After cooling to the room temperature, the mixture

was extracted with dichloromethane, and the solvent was removed, the organic layer was dried with $MgSO_4$ and the solvent was removed under vacuum. Compound Ⅱ was obtained by column chromatography on silica gel using petroleum ether and dichloromethane as eluent. The product was dried under vacuum to give Ⅱ as white solid with about 70% yield. ^{1}H NMR of Ⅱ (400 MHz, Chloroform-d) δ = 7.08 (d, 8H), 6.77 (d, 8H), 3.95 (t, 8H), 3.43 (t, 8H), 1.93-1.74 (m, 16H), 1.55-1.20 (m, 64H).

Synthesis of compound Ⅲ: Compound Ⅱ (0.1 mmol), K_2CO_3 (2.0 mmol), KI (1.0 mmol), Y6 core (0.5 mmol) were mixed in DMF (10.0 ml) to stir at 75 ℃ for 24 h under argon atmosphere. After that, 2-ethylhexyl bromide (1.0 mmol) was added and the mixture was stirred for another 12 h. After cooling to the room temperature, the mixture was extracted with dichloromethane. The organic layer was dried with $MgSO_4$ and the solvent was removed under vacuum. Compound Ⅲ was obtained by column chromatography on silica gel using petroleum ether and dichloromethane as eluent. The product was dried under vacuum to give Ⅲ as red solid with about 40% yield. ^{1}H NMR of Ⅲ (400 MHz, Chloroform-d) δ = 7.09-7.03 (d, 8H), 7.01 (s, 8H), 6.79-6.72 (d, 8H), 4.68-4.56 (m, 16H), 3.95-3.86 (t, 8H), 2.89-2.76 (t, 16H), 2.14-2.00 (m, 6H), 1.97-1.80 (m, 24H), 1.80-0.49 (m, 278H).

Synthesis of compound Ⅳ: Phosphorus oxychloride (0.5 ml) was added by injector to DMF (1.0 ml) in a two-necked flask, and the mixture was stirred for 30 min at 0 ℃ under argon atmosphere. The mixture was then transferred to a solution of compound Ⅲ (0.1 mmol) with 50.0 ml dichloroethane in another two-necked flask at 0 ℃, and refluxed at 90 ℃ overnight under argon atmosphere. The mixture was washed with saturated aqueous solution of sodium carbonate and extracted by dichloromethane. The organic layer dried with $MgSO_4$ and the solvent was removed under vacuum. Compound Ⅳ was obtained by column chromatography in a silica gel column using

dichloromethane as eluent. The product was dried under vacuum to give Ⅳ as dark red oil with about 90% yield. ^{1}H NMR of Ⅳ (400 MHz, Chloroform-d) δ = 10.14 (d, 8H), 7.05 (d, 8H), 6.75 (d, 8H), 4.75-4.55 (m, 16H), 3.91 (t, 8H), 3.18 (t, 16H), 2.13-1.83 (m, 32H), 1.74 (m, 12H), 1.59-0.55 (m, 264H).

Synthesis of GTA: Compound Ⅳ (0.1 mmol) and 2F-IC (0.9 mmol) were dissolved in toluene (5.0 ml). $BF_3 \cdot OEt_2$ (0.5 mmol) and acetic anhydride (0.1 ml) were added, and the reaction mixture was stirred at room temperature for 15 min. Then, the reaction mixture was added dropwise into methanol with stirring. The precipitate was collected, washed with methanol several times, then dissolved in chloroform and added dropwise into methanol again to afford crude product. This process was repeated several times until pure enough for photovoltaic devices. ^{1}H NMR of GTA (400 MHz, Chloroform-d) δ = 8.78 (s, 4H), 8.49 (s, 4H), 8.45(dd, 8H), 8.35 (dd, 8H), 7.56 (dt, 8H), 7.08 (d, 8H), 6.74 (d, 8H), 4.68 (d, 16H), 3.93 (t, 8H), 2.91 (s, 16H), 2.48 (s, 8H), 2.22-0.42 (m, 292H). ^{13}C NMR of GTA (100 MHz, $CDCl_3$, δ): 185.85, 165.40, 157.90, 157.52, 155.56, 155.41, 155.28, 153.79, 153.54, 152.99, 152.91, 152.84, 152.79, 152.70, 152.65, 147.18, 146.93, 145.16, 145.04, 137.15, 137.12, 136.41, 136.35, 136.26, 136.18, 135.25, 134.75, 134.24, 133.93, 133.47, 132.94, 132.64, 132.50, 130.47, 130.29, 129.25, 119.62, 119.38, 114.75, 114.66, 114.36, 113.48, 113.25, 112.36, 112.17, 112.03, 111.85, 77.25, 68.62, 68.55, 65.43, 55.26, 51.69, 40.62, 31.95, 31.49, 31.01, 30.93, 29.88, 29.78, 29.69, 29.67, 29.58, 29.55, 29.47, 29.45, 29.38, 29.22, 29.12, 28.51, 27.91, 26.84, 25.98, 23.36, 22.87, 22.72, 14.17, 13.81, 10.41. MS (MALDI-TOF) of GTA *m/z*: $[M + H]^{+}$ calcd for $C_{369}H_{388}F_{16}N_{32}O_{12}S_{20}$, 6406.50, found: 6396.48.

For the synthetic routes of GTA, the main lower-yield step is the synthesis of compound Ⅲ, with the yield of 40 %. However, according to the optimized reaction conditions, the by-products of this step are mainly the intermediates that can be used to synthesize Y6. Therefore, the overall yield of

the GTA molecules can be calculated as 70 %* 90 % * 90 % * 95 % to be about 54 %.

Material characterization

NMR: ^{1}H NMR and ^{13}C NMR were recorded on Bruker AVANCE 400 MHz NMR spectrometer with $CDCl_3$ as solvent.

MS: MALDI-TOF mass spectrometry experiments were performed on an autoflex III instrument (Bruker Daltonics, Inc.).

TGA: TGA was measured on HTG-1 Thermogravimetric Analyzer (Beijing Hengjiu Experiment Equipment Co. Ltd.) with a heating rate of 10 ℃ min^{-1} under a nitrogen flow rate of 100 ml min^{-1}.

DSC: DSC measurements were performed on a Mettler Toledo DSC1 star system, the heating rate and cooling rate were both kept 10 ℃ min^{-1} under a nitrogen flow rate of 75 ml min^{-1}. The samples were loaded in aluminum pans directly with another empty aluminum pan as the reference. As for the blend samples, donor and acceptor materials were solved in chloroform (20 mg ml^{-1} for acceptor, gradient proportion of donor by mass concentration) and stirred overnight. Next, the solution was spin-coated onto cleaned glass substrates and dried under vacuum to form homogeneous films. The samples were then scraped off the substrates and loaded in aluminum pans.

Cyclic voltammetry: Cyclic voltammetry was conducted on a Zahner IM6e electrochemical workstation using sample films coated on glassy carbon as the working electrode, Pt wire as the counter electrode, and Ag/AgCl as the reference electrode, in a 0.1 M tetrabutylammonium hexafluorophosphate (Bu_4NPF_6) acetonitrile solution and ferrocene/ferrocenium (Fc/Fc^+) couple was used as an internal reference. From the onset oxidation potential (φ_{ox}) and onset reduction potential (φ_{red}), the highest occupied molecular orbital (HOMO) energy level (E_{HOMO}) and the lowest unoccupied molecular orbital (LUMO) energy level (E_{LUMO}) of the acceptors are calculated

according to the equation of $E_{LUMO/HOMO}$ = -e ($\varphi_{red/ox}$ + 4.8 - $\varphi_{Fc/Fc+}$) (eV), where the unit of $\varphi_{red/ox}$ is V *vs*. Ag/AgCl and $\varphi_{Fc/Fc+}$ is 0.434 V *vs*. Ag/AgCl in our measurement system.

UV-visible absorption: The UV-vis absorption spectra were measured by Hitachi U-2910 UV-vis spectrophotometer. The dilute solution of acceptors in chloroform (1×10^{-5} M) was prepared to be measured, while the thin films of acceptors were prepared by spin-coating (3000 rpm) chloroform solution (10 mg ml^{-1}) on quartz plates with the thickness ranging from 50 nm to 80 nm, recorded on Bruker DEKTAK XT step profiler. For the evaluation of T_g, the thin films were annealed at various temperatures (25 ℃ or 160 ℃) for 5 mins in air after spin-coating.

***in-situ* UV-vis absorption:** The *in-situ* absorption spectra were measured by RU-200 in situ UV-vis spectrophotometer (Shaanxi Puguang Weishi Technology Co., Ltd). The time interval of spectral sampling was 0.2 s, and transmission mode is used in the collection of *in-situ* spectroscopy signal. The pure films of acceptors were spin-coated on ITO with a concentration of 8.73 mg/mL, while the blends films are with the D:A ratio of 1:1.2 and the concentrations of 16 mg/mL. Absorption baseline was taken with PET substrate before each coating session.

AFM: Atomic force microscope (AFM) measurements were conducted with a Dimension Icon2-SYS AFM instrument (Bruker) in the tapping mode. All film samples were spin-cast on ITO substrates.

TEM: Transmission electron microscope (TEM) measurements were conducted with a FEI Tecnai G2 F20 electron microscopy.

GIWAXS: The GIWAXS measurements of single and blend films were conducted at Beijing Synchrotron Radiation Facility (BSRF) beamline 1W1A.

DMA: The DMA were conducted with a Mettler Toledo, with the heating rate of 5 ℃/min, the temperature range is -100-100 ℃, and the frequency is 5 Hz. The test samples were drop-casted

onto thin continuous polyimide substrates, dried overnight at room temperature and then annealed at 80 ℃ to eliminate thermal history, and the templates were used to prepare test specimens with a size of 30×5 mm.

Calculation methods of the Flory-Huggins interaction parameters (χ): The Flory-Huggins interaction parameters (χ) of different blend films are evaluated via the T_m depression method of acceptors in homogeneous D: A mixtures with various D: A weight ratio. The related calculation equation was developed by Nishi and Wang[60-64], as shown below:

$$\frac{1}{T_m}-\frac{1}{T_m^0}=-\frac{Rv_2}{v_1\Delta H_f}[\frac{\ln\varphi_2}{m_2}+(\frac{1}{m_2}-\frac{1}{m_1})(1-\varphi_2)+\chi(1-\varphi_2)^2] \quad (1)$$

In Equation 1, subscripts 1 and 2 represent amorphous donor material and crystalline acceptor material, respectively; T_m and T_m^0 are the melting points of the D:A mixtures and the pure crystalline acceptors; ΔH_f represents the heat of fusion of the crystalline acceptors; R is the ideal gas constant; v_1 and v_2 represent the molar volumes; m is the degree of polymerization; and φ is the volume fraction. In this work, subscripts 1 and 2 represent PM6 and SMAs, respectively. For the PM6: acceptors mixtures, since the degree of polymerization of PM6 is over large compared to acceptors, m_1 can be seen as ∞ and m_2 to be 1, so that Equation 1 can be simplified as:

$$\frac{1}{T_m}-\frac{1}{T_m^0}=-\frac{Rv_2}{v_1\Delta H_f}[\ln\varphi_2+(1-\varphi_2)+\chi(1-\varphi_2)^2] \quad (2)$$

Moreover, χ can be of the following form if neglect the effects of entropy and φ_2,

$$\chi=\frac{\beta v_1}{RT_m} \quad (3)$$

where β represents the interaction energy density characteristic of the organic material pair. By substituting Equation 3 into Equation 2, Equation 4 is obtained as below, and the linear relationship between $-[1/T_m-1/T_m^0+Rv_2(\varphi_1+\ln\varphi_2)/(\varphi_1\Delta H_f)]/\varphi_1$ and φ_1/T_m represents the corresponding χ values.

$$-\frac{1}{\varphi_1}[\frac{1}{T_m}-\frac{1}{T_m^0}+\frac{Rv_2}{v_1\Delta H_f}(\varphi_1+\ln\varphi_2)]=\frac{\beta v_2}{\Delta H_f}\cdot\frac{\varphi_1}{T_m} \quad (4)$$

Device fabrication and characterization

Device fabrication: The conventional device structure of ITO/PEDOT: PSS/active layer/PDINN/Ag was constructed. The indium tin oxide (ITO) substrates were prepared in an order of deionized water, acetone, ethanol, then dried in oven at 100 ℃ for 30 min. The substrates were treated with ultraviolet ozone for 10 min and the PEDOT: PSS aqueous solution (Baytron P 4083 from H. C. Starck) was filtered through a 0.45 mm filter and then spin-coated on precleaned ITO-coated glass at 6000 rpm for 30 s. After annealing at 150 ℃ on hot plate for 20 min, the substrates were transferred into a N2 protected glove box. All the active layers were obtained by spin-coating the chloroform solution containing D: A blend (w/w, 1:1.2) in a total concentration of 16 mg ml^{-1} with 0.8% 1-CN, and the active layers were annealed at 100 ℃ for 7 min. Subsequently, ~10 nm PDINN as cathode interlayer was spin-coated onto the active layers in a concentration of 1 mg ml^{-1} in methanol solution. Finally, about 100 nm Ag were vacuum thermally deposited on the top of the device through a shadow mask. For the thermal stressed device, to avoid the diffusion of organic cathode interlayer, inverted devices were fabricated with a device structure of glass/ITO/ZnO(20 nm)/PM6:acceptors (100 nm)/MoOx(10 nm)/Ag(100 nm). Subsequentially, 10 nm MoOx and 100 nm Ag were thermally evaporated with a shadow mask on the top of the active layer.

Device characterization: The current density-voltage (*J*-*V*) characteristic were measured by using the solar simulator (SS-F5-3A, Enlitech, Taiwan) along with AM 1.5G (100 mW cm^{-2}). The external quantum efficiency (EQE) was recorded with a QE-R measurement system (Enlitech, Taiwan). The effective area of all devices was confined as 0.06 cm^2. The hole-only and electron-only devices were fabricated with the architectures of ITO/PEDOT: PSS/active layer/MoO_3/Ag and ITO/ZnO/active layer/PDINN/Ag, respectively. Hole-only and electron-only devices were recorded with a Keithley 236 sourcemeter under dark. The hole and electron mobility were determined by

fitting the dark current to the model of single-carrier SCLC, which is described by the equation 5,

$$J = \frac{9}{8}\varepsilon_0\varepsilon_r\mu\frac{V^2}{d^3} \quad (5)$$

where J is the current density, μ is the zero-field mobility, ε_0 is the permittivity of free space, ε_r is the relative permittivity of the material, d is the thickness of the active layers, and V is the effective voltage. The effective voltage was obtained by subtracting the built-in voltage (V_{bi}) and the voltage drop (V_s) from the series resistance of the whole device except for the active layers from the applied voltage (V_{appl}), $V = V_{appl} - V_{bi} - V_s$. The hole and electron mobilities can be calculated from the slope of the $J^{1/2}$-V curves.

Photo-stability characterization: The long-term stability of encapsulated devices was evaluated using multi-channel solar cell performance decay test system (PVLT-6001M-32A, Suzhou D&R Instruments Co. Ltd.). In our test, the glass-encapsulated devices were exposed to continuous white LED light (D&R Light, L-W5300KA-150, Suzhou D&R Instruments Co. Ltd.) while being stored in air. The initial illumination intensity was adjusted to match the J_{sc} measured under standard conditions by AM1.5G. During the test, the Illumination intensity was monitored using a photodiode. Periodic J-V characterization of the devices allowed for the calculation of photovoltaic parameters, including V_{oc}, J_{sc}, FF, and PCE according to the J-V curves.

Supplementary Figures

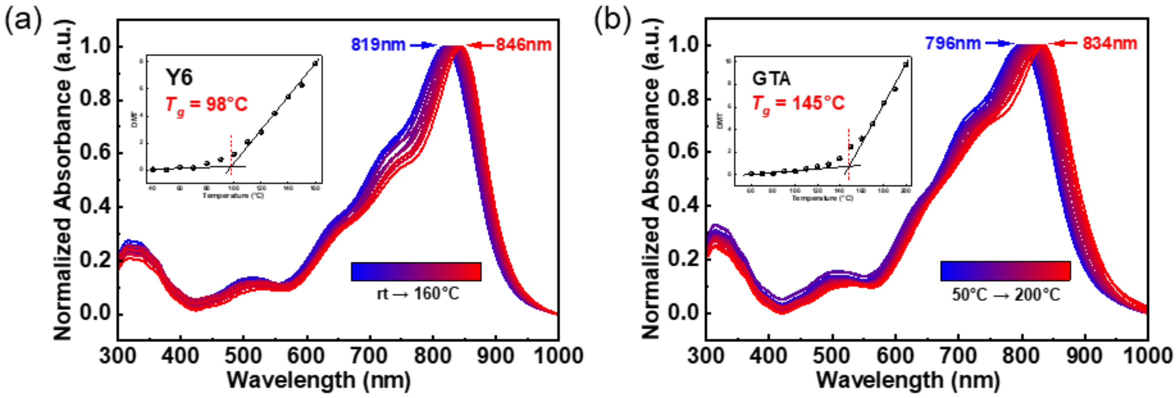

Figure S1 Normalized absorption spectra of (a) Y6 and (b) GTA thin films thermal annealing at varied temperatures, with the corresponding deviation metric and T_g values inserted.

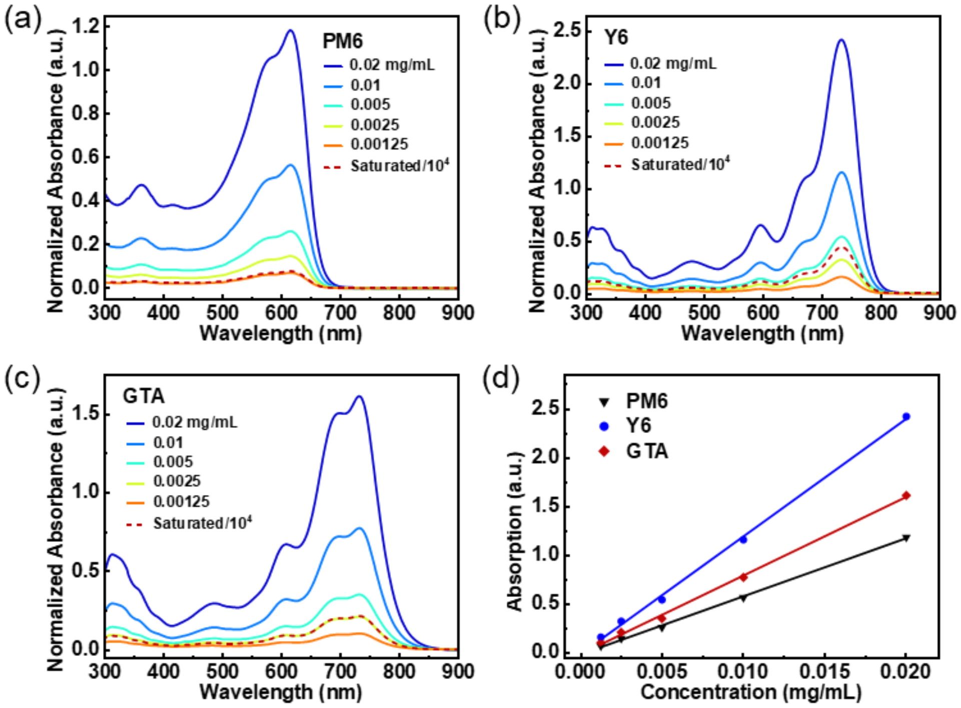

Figure S2 Absorbance in diluted chloroform solutions with different concentrations for (a) PM6, (b) Y6, (c) GTA. (d) The corresponding relationship between absorbance and concentration of solution.

The solubility of the PM6, Y6 and GTA were measured based on Lambert-Beer law (*Small.*, 2024, 2405415; *Nat Energy.* 2021; 6(11): 1045-1053), to be 15.7 mg mL^{-1}, 37.9 mg mL^{-1} and 28.4 mg mL^{-1}, respectively

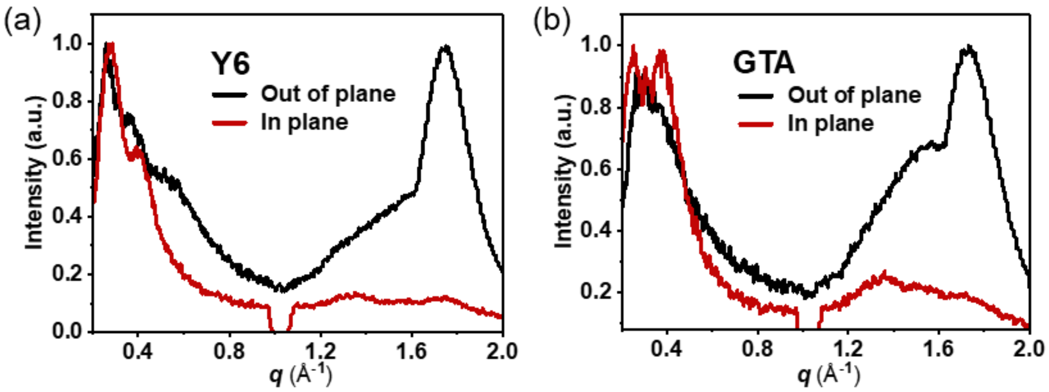

(c)

Acceptors	*d* [100] (Å)	CCL[100] (Å)	*d* [010] (Å)	CCL[010] (Å)	g (%)
Y6	22.12	43.84	3.59	30.73	13.6
GTA	25.23	64.26	3.63	35.57	12.7

Figure S3 line-cuts of 2D-GIWAXS patterns in the in-plane and out-of-plane directions of (a) Y6, (b) GTA pure film after annealing at 100 ℃ for 5 min, and (c) the corresponding crystallinity properties.

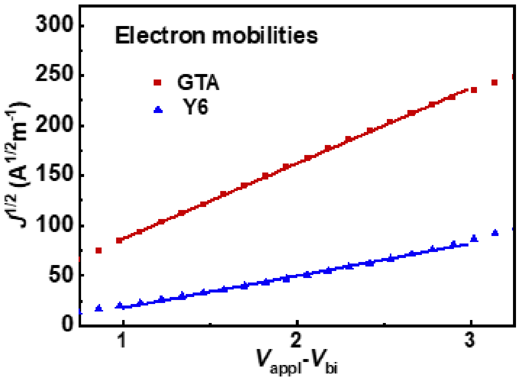

Figure S4 Electron mobilities of Y6 and GTA.

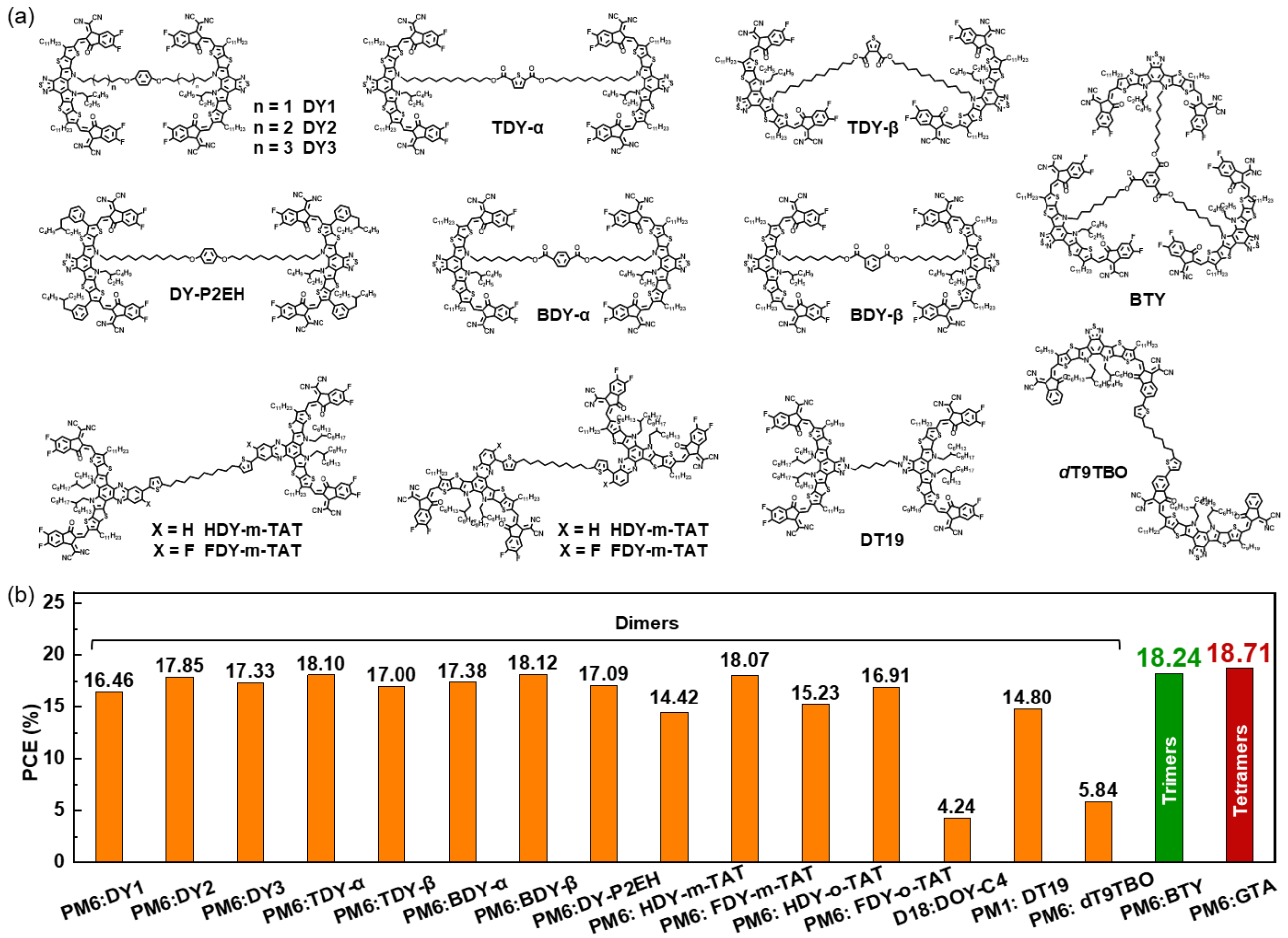

Figure S5 (a) Molecule structures of oligomeric accepters with flexible chains. (b) The statistical PCE values of corresponding binary PSCs devices.

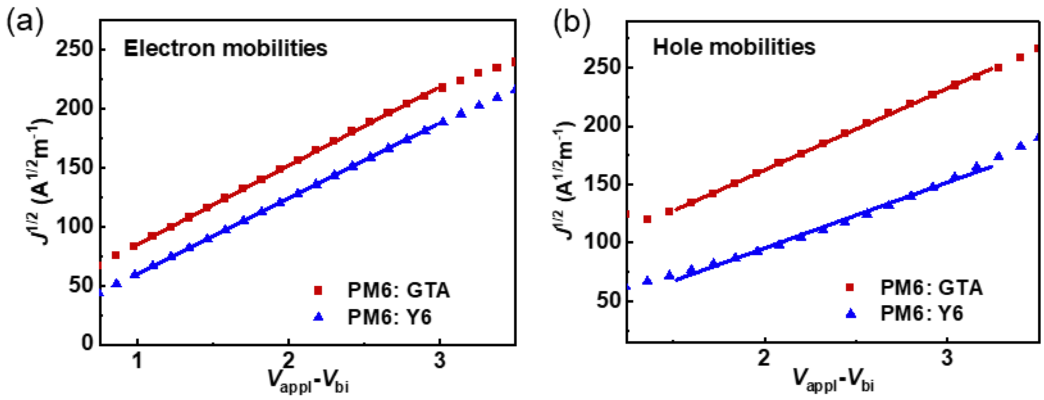

Figure S6 (a) Electron mobilities and (b) hole mobilities of blend films.

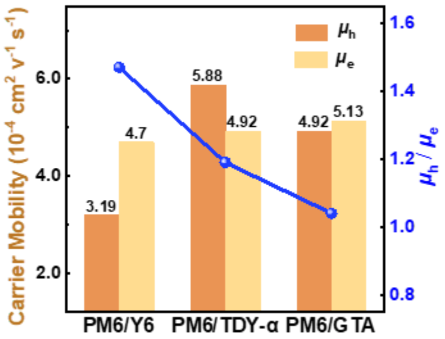

Figure S7 The hole/electron mobilities and the corresponding ratio of PM6:Y6, PM6:TDY-α and PM6:GTA devices.

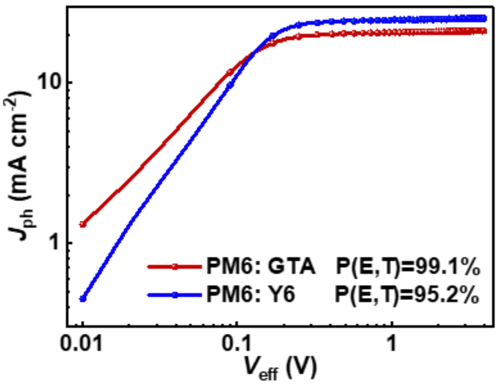

Figure S8 J_{ph} versus V_{eff} of the optimized devices.

To gain insights on exciton dissociation and charge collection behavior of the PSCs, the charge dissociation probability (P(E,T)) is estimated with relationship between photo current density (J_{ph}) and the effective voltage (V_{eff}) of the TSMAs-based devices. The photocurrent density J_{ph} is defined as $J_{ph} = J_L - J_D$, where J_L and J_D are the photocurrent densities under AM 1.5G illumination and in the dark condition, respectively. The effective voltage V_{eff} is defined as $V_{eff} = V_0 - V_{bias}$, where V_0 is the voltage at which J_{ph} is zero and V_{bias} is the applied external voltage bias. It can be seen that, the increase in V_{eff} results in a higher internal electric field in the device; accordingly, the charge recombination will be minimized. In our cases, J_{ph} tends to an approximate saturation value (J_{sat}) at an adequate high V_{eff} of 2.0 V. Thus, the charge dissociation probability (P(E,T)) can be estimated from equation of P(E,T) = J_{ph} / J_{sat}.

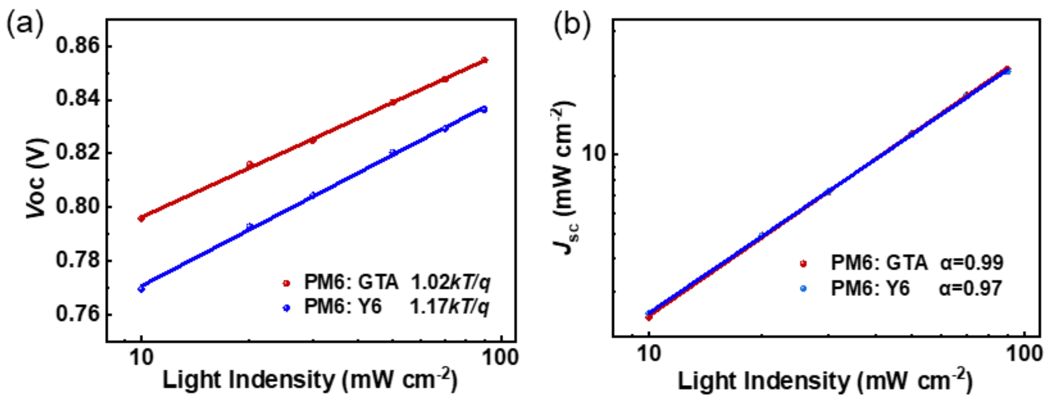

Figure S9 Light intensity dependence of V_{oc} and J_{sc} of the PSCs.

The charge recombination behavior of the PSCs is investigated by the dependence of V_{oc} and J_{sc} on light intensity (P_{light}). The degree of trap-assisted recombination can be estimated by the relationship of $V_{oc} \propto nk\mathrm{T}/q\ln(P_{light})$. Here, n represents the ideality factor, k is the Boltzmann constant, T is absolute temperature, and q is the elementary charge. If the slope is $k\mathrm{T}/q$, it suggests that bimolecular recombination is the main recombination. And if the slope is close to 2 $k\mathrm{T}/q$, it indicates serious trap-assisted recombination.

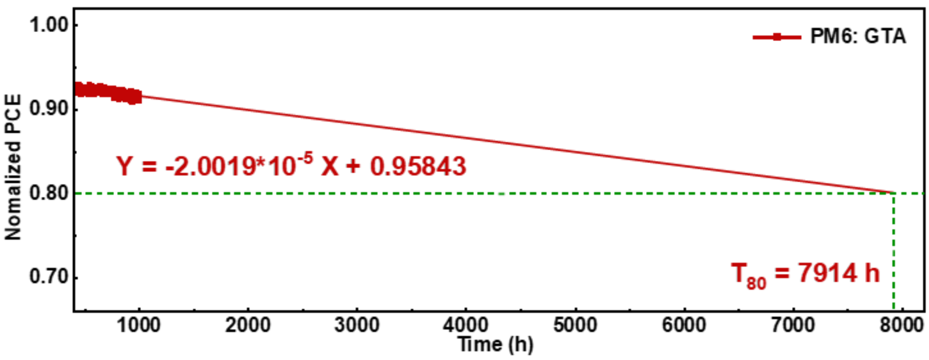

Figure S10 MPP stability test of the PM6:GTA based devices under 1-Sun equivalent illumination from white LEDs at the MPP conditions in open-air. The solid red line corresponds to the linear fitting result from 400 h to 1100 h, excluding the influence of burn-in loss.

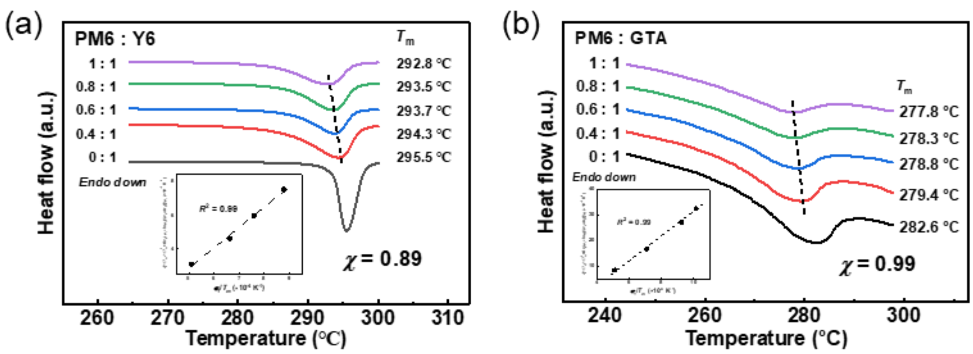

Figure S11 DSC thermograms of various (a) PM6:Y6 and (b) PM6:GTA mass ratios, and insert shows the estimates of the χ values by the melting point depression from DSC thermograms.

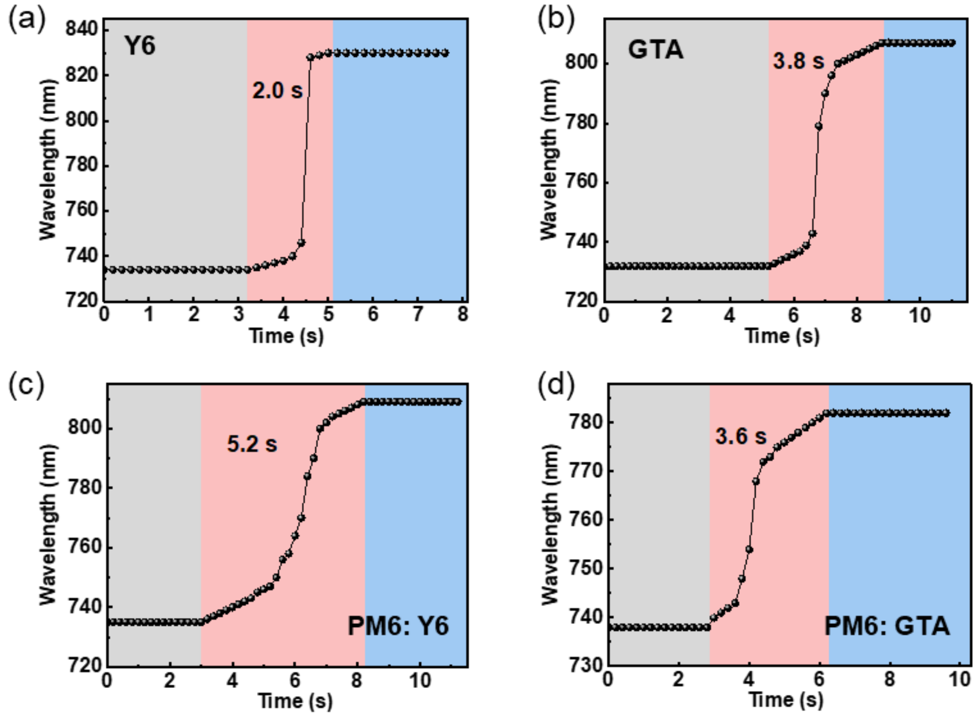

Figure S12 The *in-situ* absorption location as a function of spin-coating time.

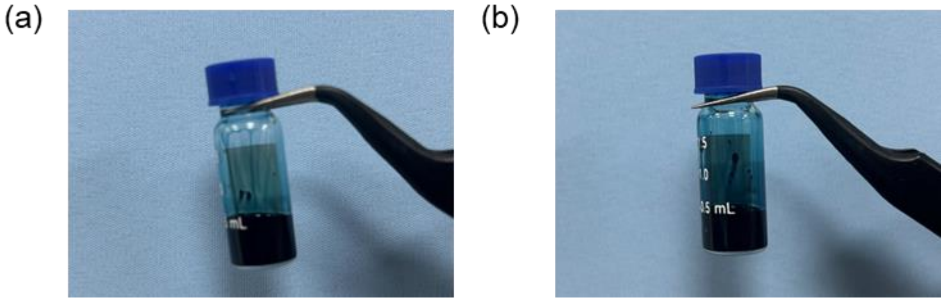

Figure S13 The solubility test photographs of (e) Y6 and (f) GTA in the saturated chloroform solution of PM6.

The solubilities of Y6 and GTA in the saturated chloroform solution of PM6 were about 40 mg mL^{-1} and 30 mg mL^{-1}, respectively.

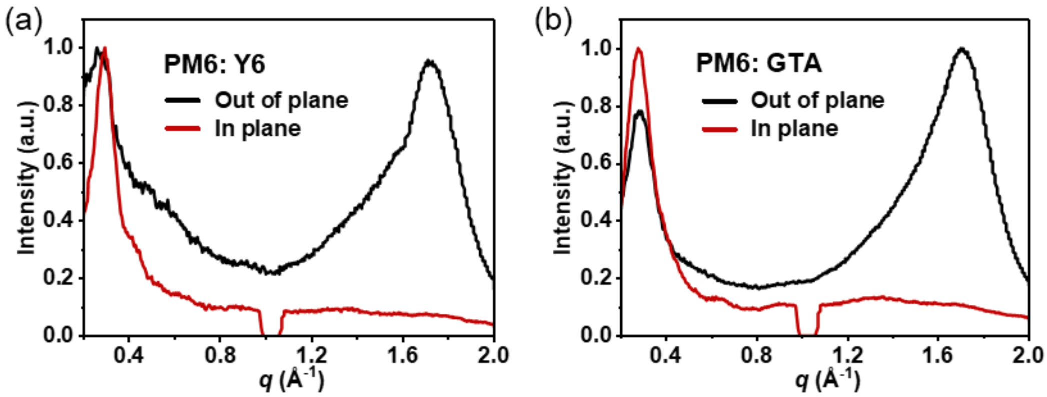

(c)

Blend film	*d* [100] (Å)	CCL[100] (Å)	*d* [010] (Å)	CCL[010] (Å)	g (%)
PM6: Y6	21.59	65.75	3.65	36.02	12.7
PM6: GTA	22.44	54.90	3.70	30.08	14.0

Figure S14 line-cuts of 2D-GIWAXS patterns in the in-plane and out-of-plane directions of (a) PM6:Y6, (b) PM6:GTA blend film after annealing at 100 ℃ for 5 min, and (c) the corresponding crystallinity properties.

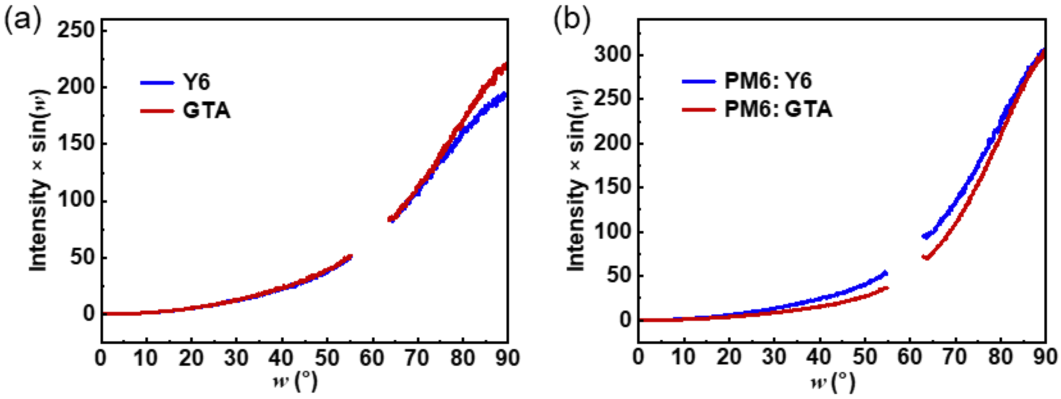

Figure S15 The calculation of the relative degree of crystallinity (*r*DoC), calculated via the azimuthal distributions of (010) diffraction peaks according to the GIWAXS.

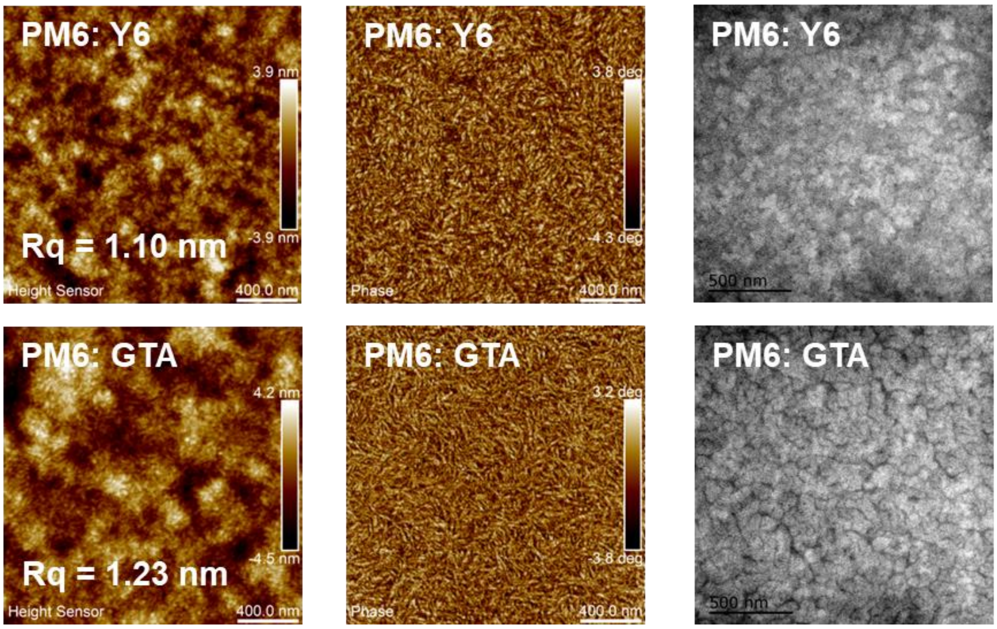

Figure S16 AFM height images, phase images and TEM images of the PM6:Y6 and PM6:GTA blend films.

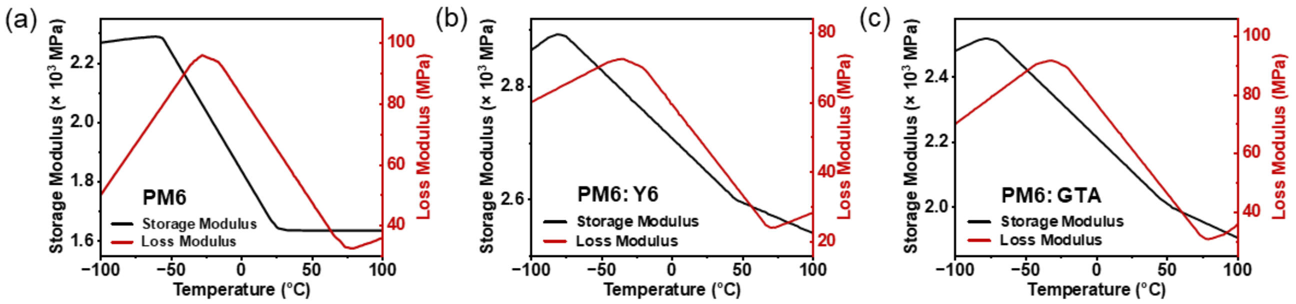

Figure S17 Storage modulus and loss modulus versus temperature of (a) PM6, (b) PM6:Y6, and (c) PM6:GTA, respectively.

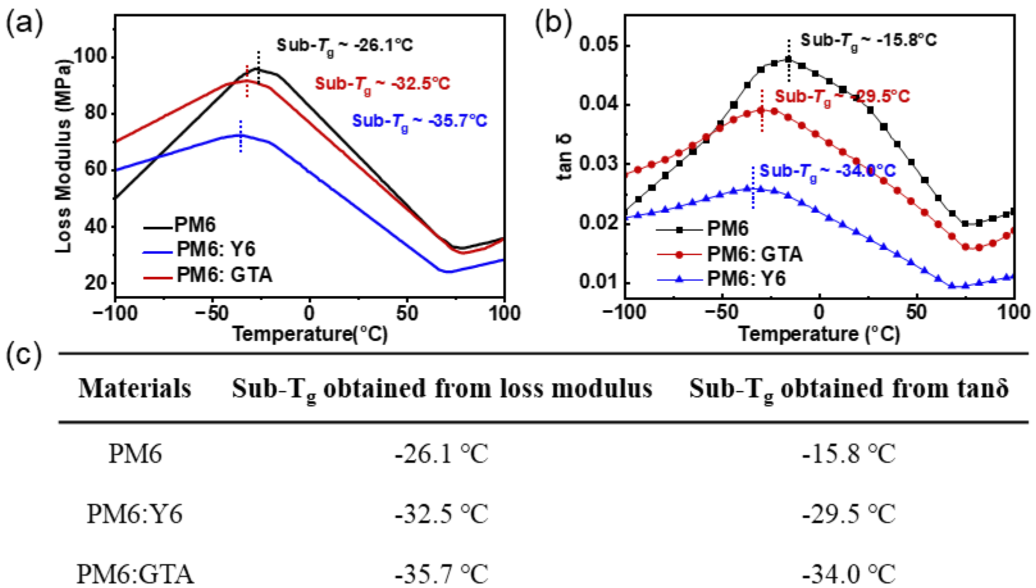

(c)

Materials	Sub-T_g obtained from loss modulus	Sub-T_g obtained from tanδ
PM6	-26.1 °C	-15.8 °C
PM6:Y6	-32.5 °C	-29.5 °C
PM6:GTA	-35.7 °C	-34.0 °C

Figure S18 The comparison of curves of (a) loss modulus and (b) tanδ versus temperature of PM6, PM6:Y6, and PM6:GTA, with the corresponding sub-T_g value labeled. (c) the statistical sub-T_g values.

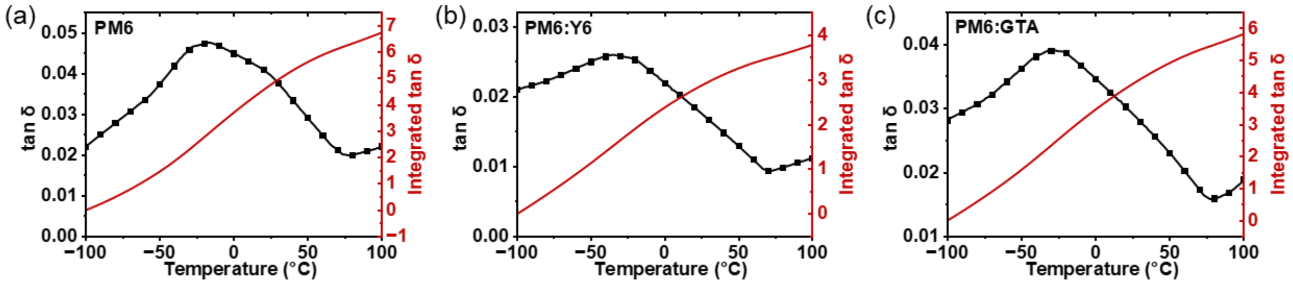

Figure S19 The tanδ and integrated tanδ ($I_{\tan\delta}$) curves of PM6, PM6:Y6, and PM6:GTA.

The strength of the sub-T_g relaxation ($I_{\tan\delta}$) was quantified by integrating sub-T_g tanδ over the temperature range, according to following equation:

$$I_{tan\,\delta} = \int_{T\,=\,-100}^{T\,=\,100} f(tan\,\delta\,,T)\,dT$$

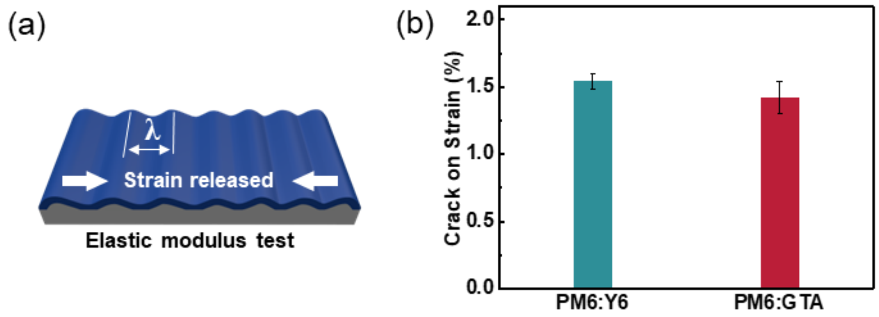

Figure S20 (a) The illustration of elastic modulus test. (b) The elastic modulus of the PM6:Y6 and PM6:GTA blend films.

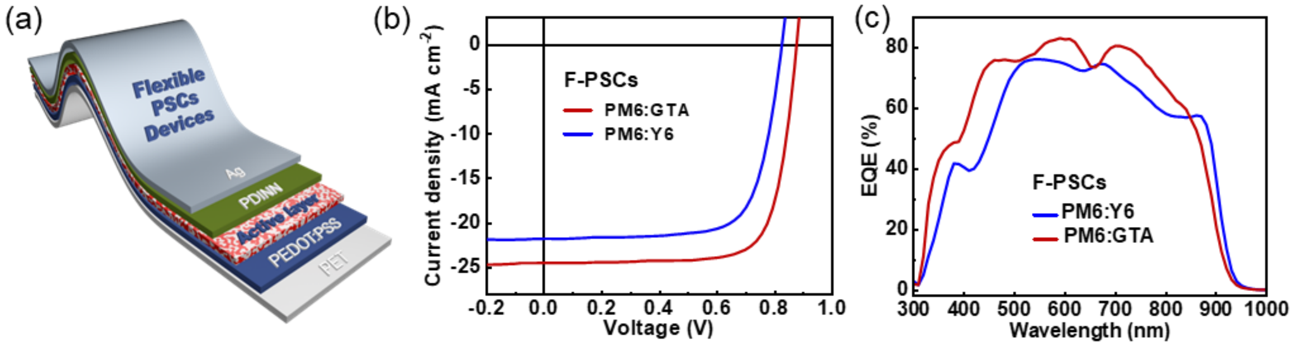

Figure S21 (a) Device structure of flexible PSCs. (b) *J-V* curves of the flexible PSCs and (c) EQE spectra of corresponding F-PSCs.

The intrinsically stretchable PSCs were fabricated with the device architecture of EGaln/PNDIT-F3N/active layer/PH1000/Al4083/TPU.

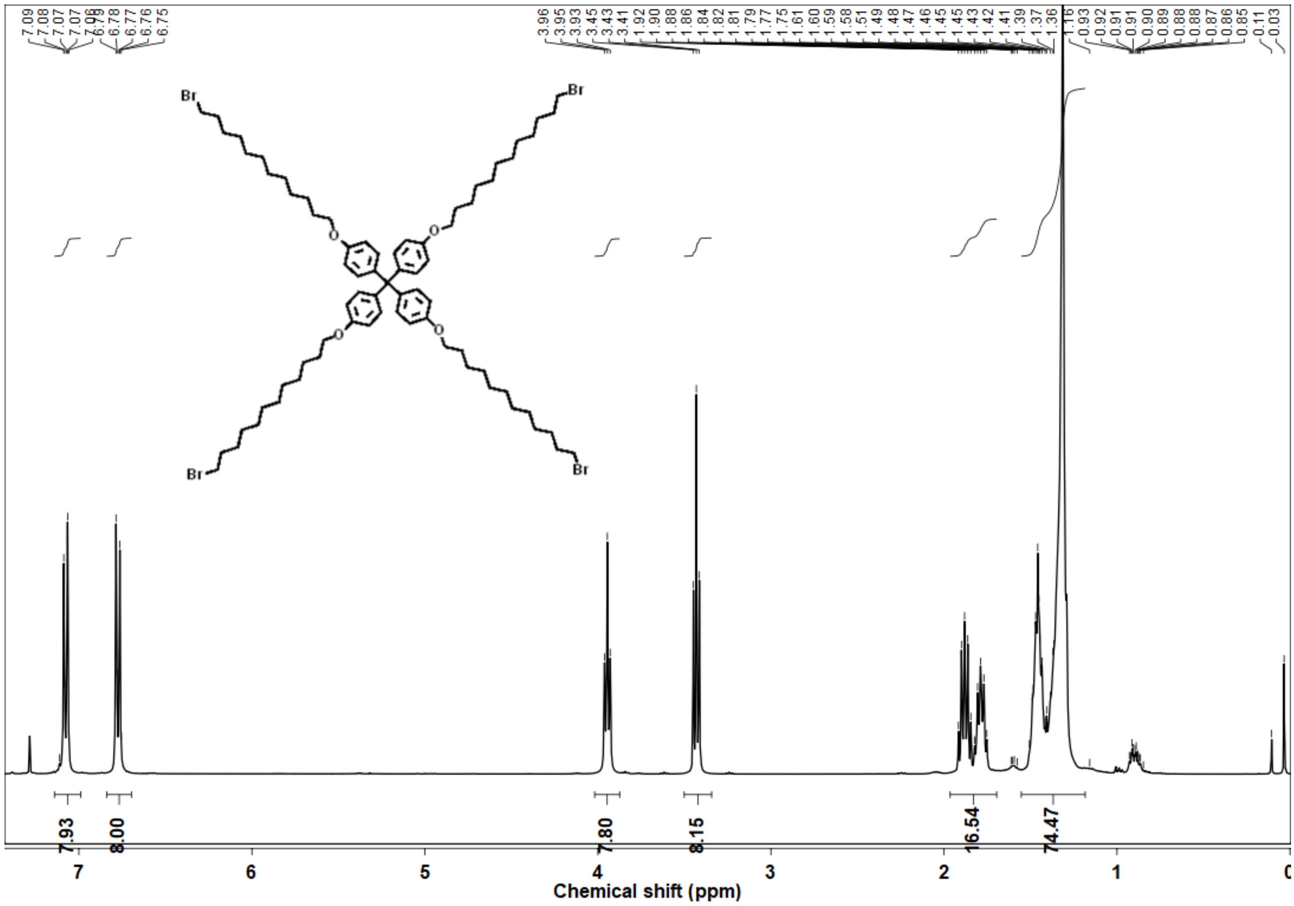

Figure S22 ^{1}H NMR spectrum of Ⅱ.

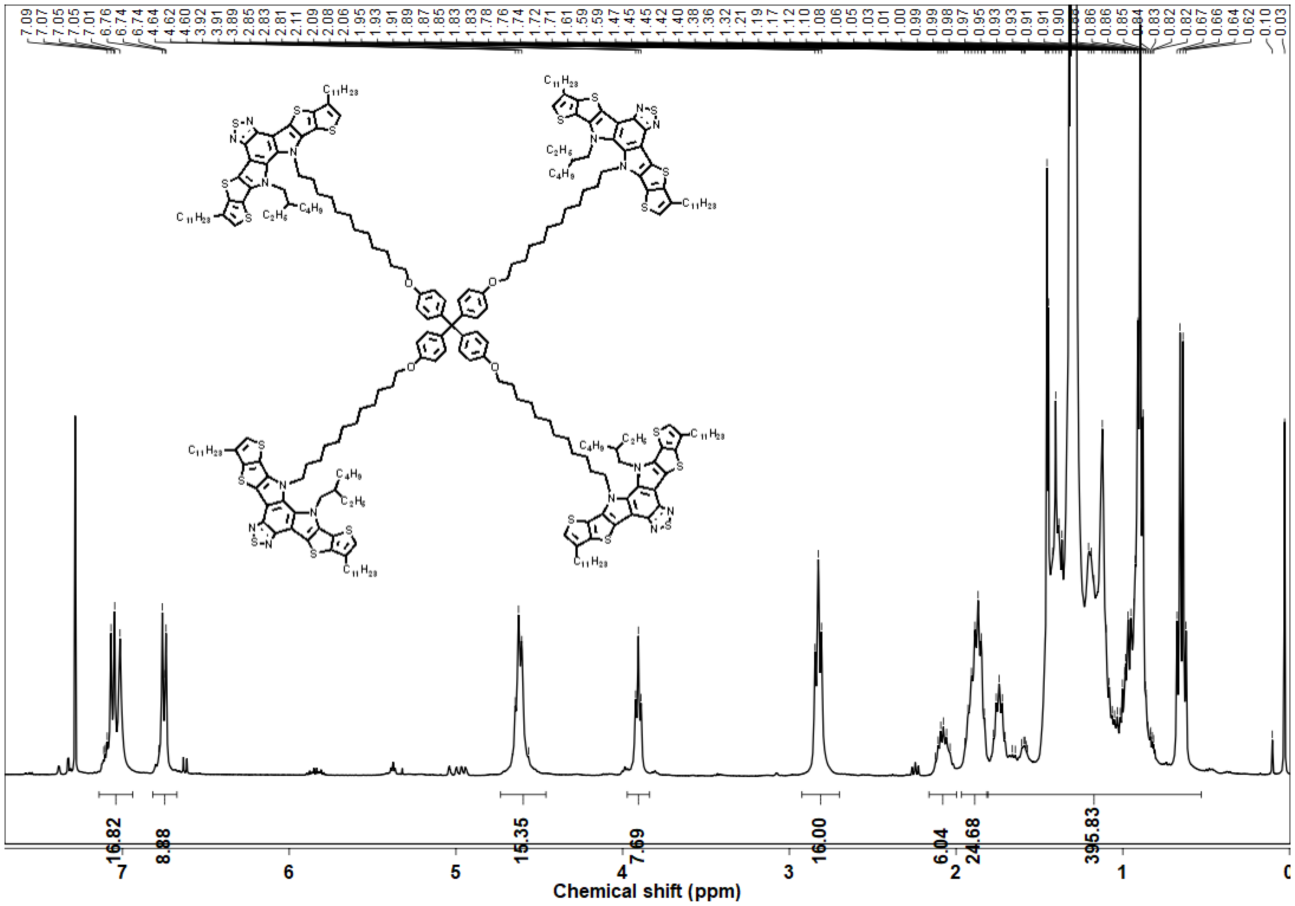

Figure S23 ^{1}H NMR spectrum of Ⅲ.

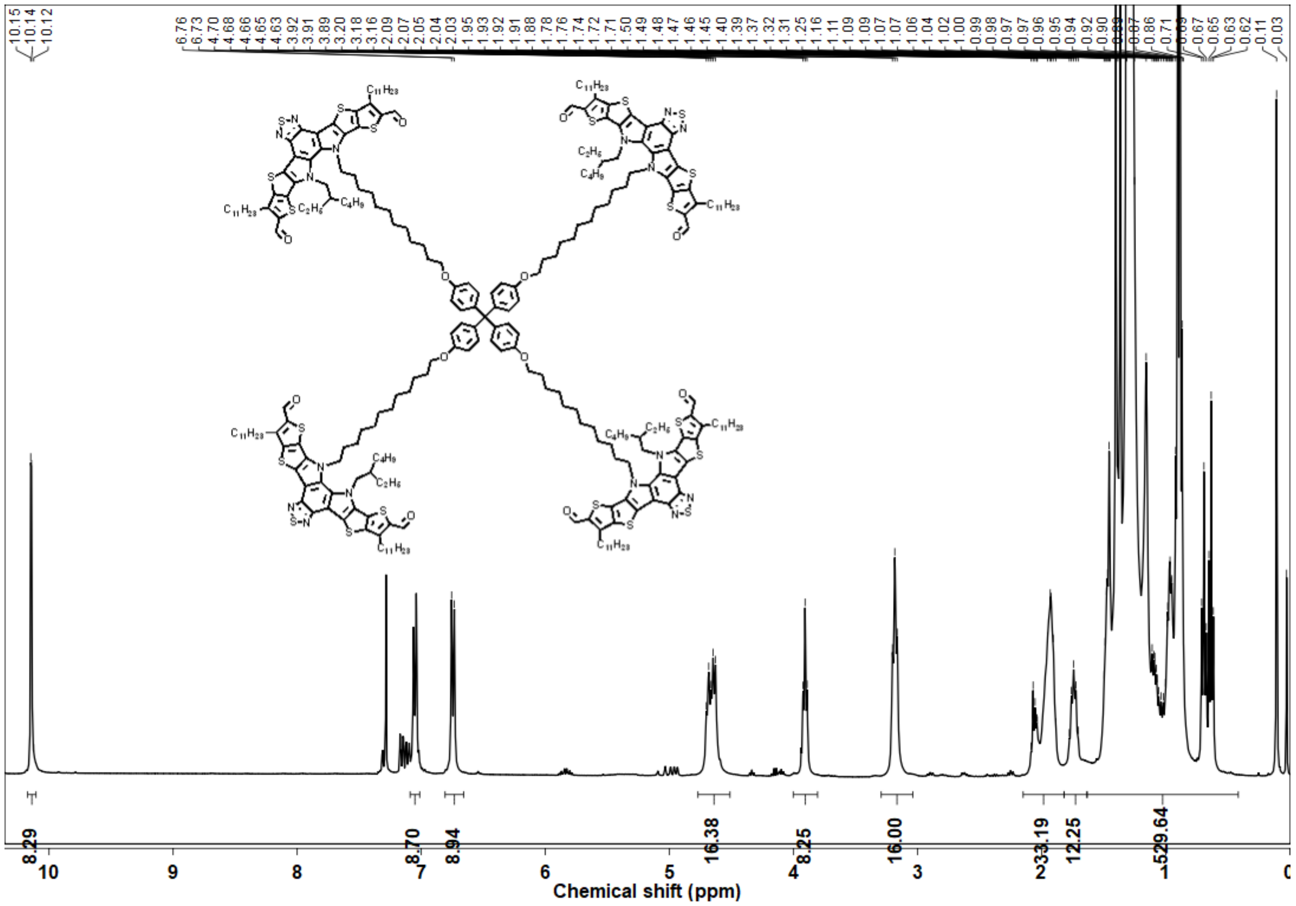

Figure S24 ^{1}H NMR spectrum of Ⅳ.

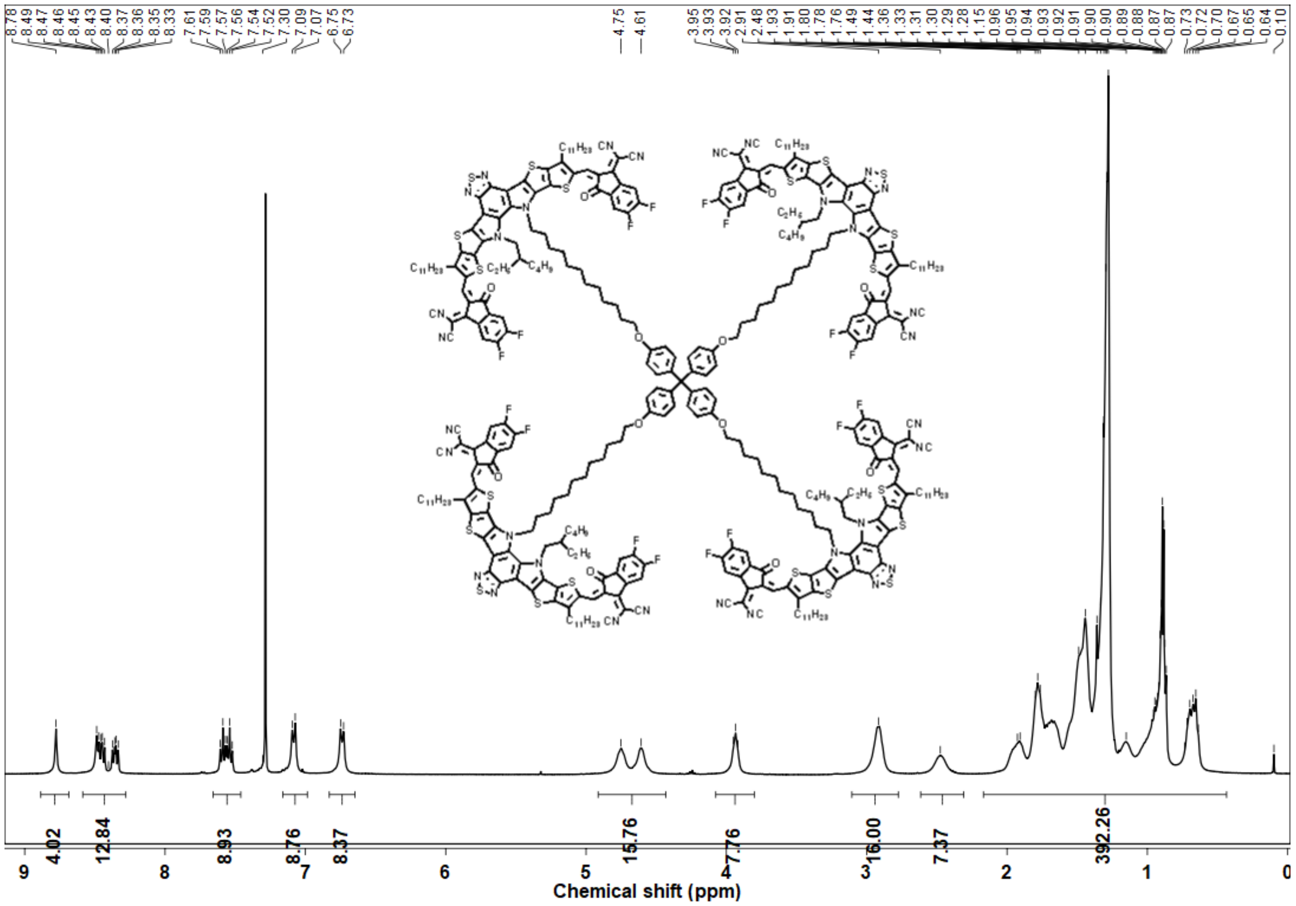

Figure S25 ^{1}H NMR spectrum of GTA.

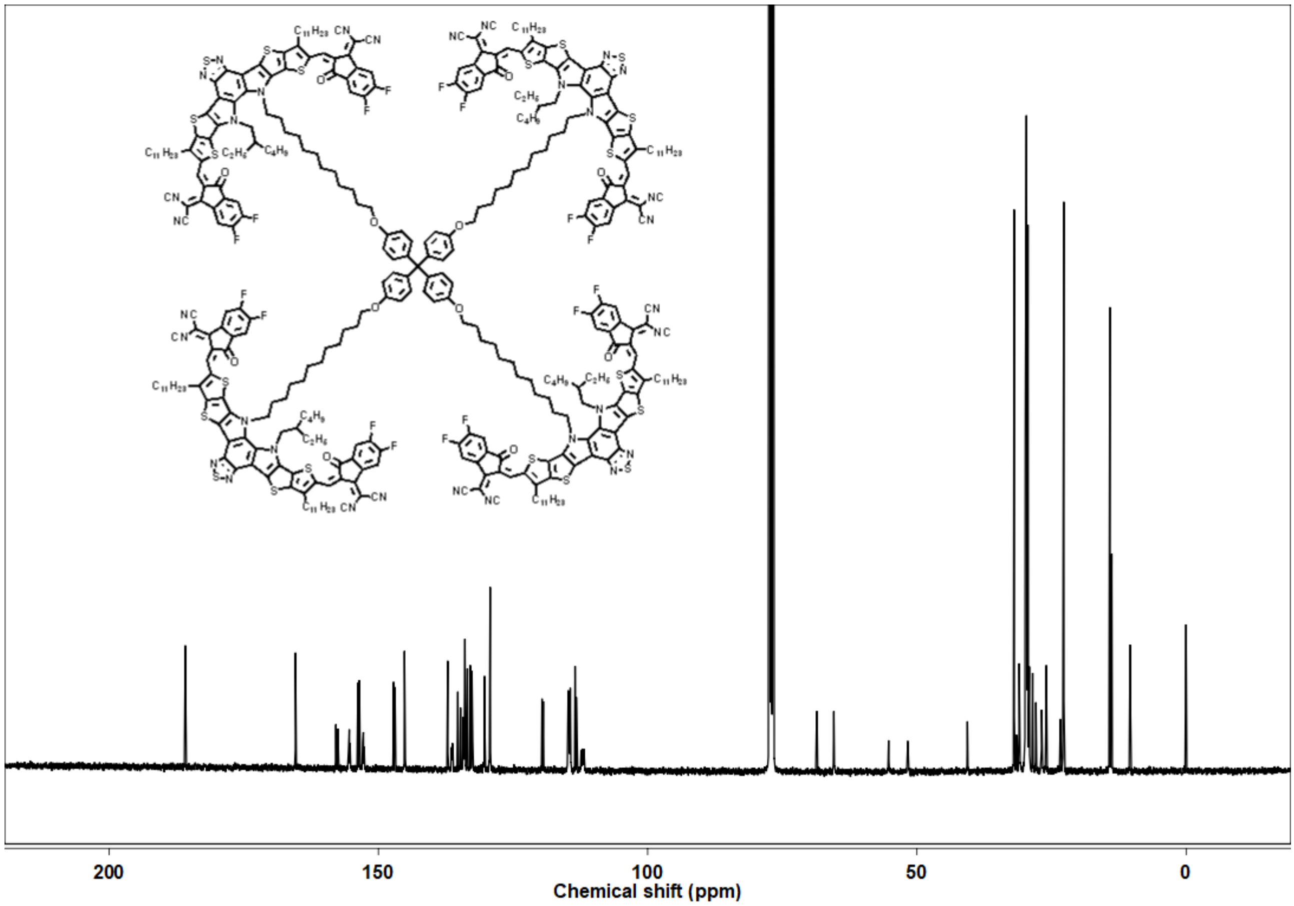

Figure S26 ^{13}C NMR spectrum of GTA.

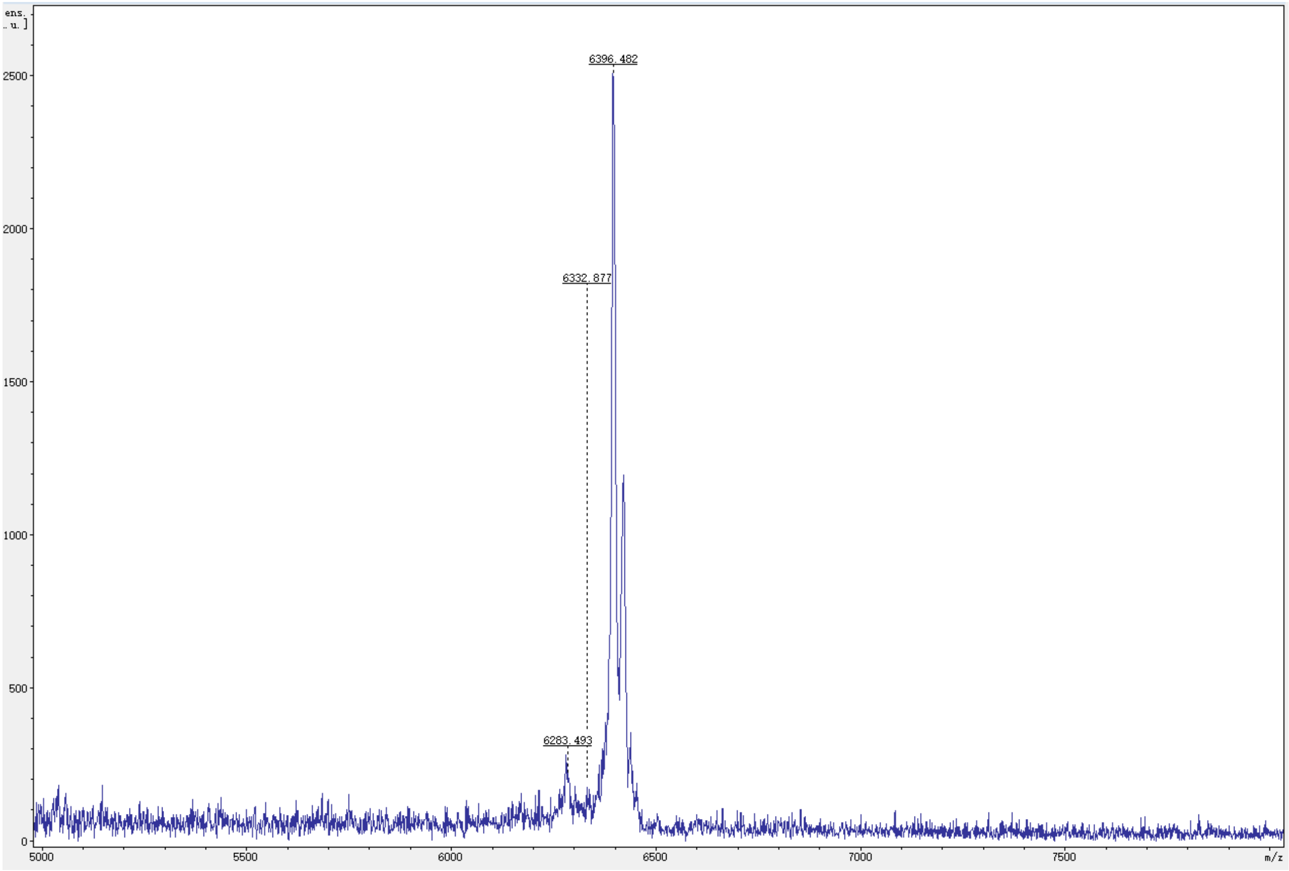

Figure S27 MS (MALDI-TOF) spectrum of GTA.

Supplementary Table

Table S1 PCE values of binary PSCs based on oligomeric accepters with flexible chains in previous work and our work.

Active layer	PCE (%)	Ref.: DOI
PM6:DY1	16.46	
PM6:DY2	17.85	10.1002/adma.202206563
PM6:DY3	17.33	
PM6:TDY-α	18.10	10.1038/s41467-023-38673-5
PM6:TDY-β	17.00	
PM6:BDY-α	17.38	
PM6:BDY-β	18.12	10.1002/anie.202400590
PM6:BTY	18.24	
PM6:DY-P2EH	17.09	10.1002/adma.202308606
PM6:HDY-m-TAT	14.42	
PM6:FDY-m-TAT	18.07	10.1002/anie.202403139
PM6:HDY-o-TAT	15.23	
PM6:FDY-o-TAT	16.91	
D18:DOY-C4	4.24	10.1002/adma.202305562
PM1:DT19	14.8	10.1002/adma.202302592
PM6:*d*T9TBO	5.84	10.1002/anie.202303066

Table S2 PCE and COS of binary PSCs in previous work and our work.

Active layer	PCE (%)	COS (%)	Ref.: DOI
PM6:PYF-IT	15.02	5.9	10.1039/D3EE01683H
PM6:PY-IT	16.59	6.5	
PTQ10:PY-IT	13.69	3.98	10.1002/adma.202212275
QM-Cl:PY-IT	17.78	7.16	
PBDB-TF:PY-IT	16.7	6.3	10.1002/adma.202208926.
PBQx-TF:PY-IT	17	5.2	
PM6:PY-IT	15	5.2	10.1002/cjoc.202200564
PM6:PYF-IT	15.1	6.3	
PM6:PY-IT	15.49	9.67	10.1002/adma.202208008
PM6:PYTCl-A	16.16	17.2	
PM6-A:PYTCl-A	15.54	20.01	
PM6-B:PYTCl-A	14.03	18.55	
PM6:PYTCl-B	13.61	18.67	
PM6-A:PYTCl-B	13.29	21.02	
PM6-B:PYTCl-B	12.73	22.74	
PBDB-TF:PY-IT	15.8	5.3	10.1002/adma.202205009
PQM-Cl:PY-IT	18	6.5	
PM6:L8-BO	19.06	5.67	10.1002/adma.202307280
D18:N3	17.06	7.8	10.1002/adma.202305562.
D18:N3	18.23	7.5	10.1002/anie.202310034
PM6:CH-D1	16.62	23.1	10.1002/aenm.202300301
PM6:N3	16.3	7.2	10.1039/D2TA10091F
PM6:BTP-eC9	15.68	7	10.1016/j.matt.2022.03.012
PM6:N3	15.4	7.3	10.1002/adma.202207884
PM6:PY-IT	15.56	9.5	10.1016/j.matt.2021.12.002
J71:PY-IT	13.43	2.7	
PM6:PY-IT	16.59	9.6	10.1002/agt2.308

Table S3 Photovoltaic parameters of the flexible PSCs based on PM6:acceptor with D:A weight ratio of 1:1.2 and thermal annealing at 100 ℃ for 5 min. under the illumination of AM1.5G, 100 mW cm^{-2}.[a]

Active layer	V_{oc} (V)	J_{sc} (mA cm^{-2})	FF (%)	PCE (%)
PM6:Y6	0.833	23.83	72.39	14.45 (14.12 ±0.24)
PM6:GTA	0.878	24.76	75.59	16.43 (16.21 ±0.16)

[a] Average values based on ten devices.
